# Thyroid hormone-dependent development of early cortical networks: temporal specificity and the contribution of trkB and mTOR pathways

**DOI:** 10.3389/fncel.2013.00121

**Published:** 2013-08-06

**Authors:** Sören Westerholz, Ana D. de Lima, Thomas Voigt

**Affiliations:** ^1^Institute of Physiology, Otto-von-Guericke UniversityMagdeburg, Germany; ^2^Center for Behavioral Brain SciencesMagdeburg, Germany

**Keywords:** GABA, interneurons, neocortex, triiodothyronine, BDNF, mTOR, KCC2, NKCC1

## Abstract

Early in neocortical network development, triiodothyronine (T3) promotes GABAergic neurons' population increase, their somatic growth and the formation of GABAergic synapses. In the presence of T3, GABAergic interneurons form longer axons and conspicuous axonal arborizations, with an increased number of putative synaptic boutons. Here we show that the increased GABAergic axonal growth is positively correlated with the proximity to non-GABAergic neurons (non-GABA). A differential innervation emerges from a T3-dependent decrease of axonal length in fields with low density of neuronal cell bodies, combined with an increased bouton formation in fields with high density of neuronal somata. T3 addition to deprived networks after the first 2 weeks of development did not rescue deficits in the GABAergic synaptic bouton distribution, or in the frequency and duration of spontaneous bursts. During the critical 2-week-period, GABAergic signaling is depolarizing as revealed by calcium imaging experiments. Interestingly, T3 enhanced the expression of the potassium-chloride cotransporter 2 (KCC2), and accelerated the developmental shift from depolarizing to hyperpolarizing GABAergic signaling in non-GABA. The T3-related increase of spontaneous network activity was remarkably reduced after blockade of either tropomyosin-receptor kinase B (trkB) or mammalian target of rapamycin (mTOR) pathways. T3-dependent increase in GABAergic neurons' soma size was mediated mainly by mTOR signaling. Conversely, the T3-dependent selective increase of GABAergic boutons near non-GABAergic cell bodies is mediated by trkB signaling only. Both trkB and mTOR signaling mediate T3-dependent reduction of the GABAergic axon extension. The circuitry context is relevant for the interaction between T3 and trkB signaling, but not for the interactions between T3 and mTOR signaling.

## Introduction

Thyroid hormone has a diverse range of actions in the development and function of the CNS and plays a prominent role in neocortical development (Gilbert et al., 2012). Thyroid hormone specifically modulates the development and function of GABAergic interneurons *in vivo* and *in vitro* (Gilbert et al., 2007; Westerholz et al., 2010). Additionally, locomotor deficiencies and anxiety following disruption of thyroid hormone signaling have been linked to alterations in GABAergic interneurons development (Guadano-Ferraz et al., 2003; Venero et al., 2005; Wallis et al., 2008). Parvalbumin-immunoreactive interneurons are the most sensitive to thyroid hormone signaling deficits (Wallis et al., 2008). Accordingly, during the early cortical network development, triiodothyronine (T3) regulates the density and neuronal growth of specific GABAergic neurons' subpopulations (Westerholz et al., 2010).

A milestone in the early neuronal network development is the appearance of spontaneous network activity characterized by synchronous bursts of action potentials and concomitant intracellular calcium transients in large groups of cells (O'Donovan, 1999; Ben-Ari et al., 2007; Blankenship and Feller, 2010). The recurrent calcium transients are driven by depolarizing actions of glutamatergic and GABAergic neurotransmission (Voigt et al., 2001; Opitz et al., 2002; Cherubini et al., 2011). T3-mediated development of GABAergic neurons *in vitro* is paralleled by an accelerated maturation of early network activity (Westerholz et al., 2010). This modulation of neuronal activity by T3 during the formation of the network explains, at least partially, the effects of the hormone on the development of GABAergic neurons (Westerholz et al., 2010).

Hypothyroidism during fetal and early postnatal period results in irreversible mental retardation and motor dysfunction (Bernal, 2007; Williams, 2008; Patel et al., 2011; Gilbert et al., 2012). A critical period for thyroid hormone signaling has been proposed, since lack of T3 during the first two postnatal weeks in rats causes severe and irreversible behavioral alterations with associated cortical, hippocampal and cerebellar malformation (Oppenheimer and Schwartz, 1997; Koibuchi and Chin, 2000; Bernal et al., 2003). Although well-documented *in vivo*, little is known about the cellular mechanisms governing this critical phase.

Depolarizing GABAergic signaling has been implicated in the early morphological and functional maturation of neuronal networks (Owens and Kriegstein, 2002; Cancedda et al., 2007; Pfeffer et al., 2009; Wang and Kriegstein, 2009). Early in postnatal development, sodium-potassium-chloride co-transporter 1 (NKCC1) expression is high and potassium-chloride co-transporter 2 (KCC2) expression is low. This expression pattern has been correlated with a depolarizing action of GABAergic signaling due to the resulting high intracellular chloride concentration (Ben-Ari et al., 2007; Blaesse et al., 2009). Later a decreased NKCC1 expression and increased KCC2 expression reduce intracellular chloride concentration, and hyperpolarizing GABA_A_ signaling can be observed. This developmental GABA switch from depolarizing to hyperpolarizing signaling represents a hallmark in network maturation by closing the period of GABA driven network oscillations and setting the onset of mature GABAergic function (Ben-Ari et al., 2007; Blaesse et al., 2009; Baltz et al., 2010; Blankenship and Feller, 2010).

In the present study we explored time-sensitive features of T3 regulation of GABAergic neurons, and signaling maturation during the network development *in vitro*. We monitored the expression of the cation-chloride co-transporters KCC2 and NKCC1 in neuronal cultures from embryonic rat neocortex, and found that aspects of the thyroid hormone-mediated development of GABAergic neurons are restricted to the early period of network formation characterized by depolarizing actions of GABAergic signaling.

Additionally, we explored possible mechanisms for T3-dependent regulation of network activity and GABAergic neurons' development. Of the many putative targets of T3, the neurotrophin BDNF stands out as a prime candidate to mediate the influence of T3 on network activity and on interneuronal development. T3 has been suggested to regulate BDNF expression (Koibuchi et al., 1999; Koibuchi and Chin, 2000; Sui and Li, 2010). BDNF depolarizes cortical neurons (Kafitz et al., 1999; Blum et al., 2002), and is a mediator of activity-dependent effects and an important regulator of interneuron development (Palizvan et al., 2004; Patz et al., 2004; Woo and Lu, 2006; Huang et al., 2007; Huang, 2009).

Another candidate for the mediation of T3 effects on GABAergic neurons is the mammalian target of rapamycin (mTOR). The serine/threonine protein kinase mTOR, as a downstream target of the PI3K/Akt signaling pathway, is an important regulator of cellular growth, metabolism and survival, and is known to regulate several intracellular processes in response to extracellular and intracellular signals. In the CNS, mTOR regulates survival, differentiation and development of neurons (for review see Swiech et al., 2008). In particular, neurite growth, cell size and synaptogenesis depend on adequate mTOR activity (Verma et al., 2005; Zeng and Zhou, 2008; Buckmaster et al., 2009; Buckmaster and Wen, 2011; Crino, 2011; Okada et al., 2011). It has been shown that T3 activates tonically the phosphatidylinositol 3-kinase (PI3K)/Akt-mTOR signaling pathway by means of thyroid hormone receptors (Moeller et al., 2006; Sui et al., 2008). Additionally, mTOR dysfunction in tuberous sclerosis 1 (TSC1) mutant is associated not only with network hyperexcitability, but also with alterations of interneuron development (Fu et al., 2012). Further, mTOR modulates the network activity of hippocampal networks (Wang et al., 2007b; Li et al., 2008), and mediates plastic changes associated with BDNF activation (Schratt et al., 2004; Inamura et al., 2005; Liao et al., 2007; Zhou et al., 2010).

## Materials and methods

### Cell culture

All experiments were carried out following the European Committees Council Directive (86/609/EEC) and approved by the local animal care committee (Landesverwaltungsamt Sachsen-Anhalt, #42502-3-133 UniMD). Neuronal cultures were prepared from rat cortices and cultured for 14 days in serum-free medium with a surrounding glial feeding layer (de Lima and Voigt, 1999; Westerholz et al., 2010). In brief, each acid cleaned cover slip was fitted to a 20-mm hole in the bottom of a 60-mm Petri dish to create compartments for separated culturing of neuronal and glial cells. Cover slips were treated overnight with poly-D-lysine (0.1 mg/ml in borate buffer pH 8.5, 36°C). Purified astroglial cells prepared from cerebral hemispheres of newborn (postnatal day 0–3) Sprague-Dawley rats were plated in the outer portion of the Petri dish bottom (300 cells/mm^2^). Neuronal cultures were prepared from cerebral cortices of embryonic Sprague-Dawley rats at embryonic day 16 (E16; day after insemination = E1). The cells were taken from the dorsolateral parts of the telencephalic vesicles (excluding hippocampal and basal telencephalic anlagen). Cells were dissociated with trypsin/EDTA and seeded onto the poly-D-lysine coated glass cover slips at a density of 300–500 cells/mm^2^ in N2 medium (75% DMEM, 25% Ham's F12 and N2 supplements from Invitrogen, Carlsbad, CA). Cultures were treated at 6 DIV (days *in vitro*) with cytosine arabinoside (Ara-C) at a final concentration of 5 μM and a third of the medium volume was then changed after 24 h. In some experiments, a complete medium change was necessary at 14 DIV to change hormone conditions (see below). In these cases, previously glia-conditioned medium was used to prevent an increase of cell death due to an excess glutamate in the freshly prepared medium. All cultures were maintained in a humidified 5% CO_2_/95% air atmosphere at 36°C for 14 DIV.

### Hormone application

Neurons were cultured for 14 days in serum-free N2 medium in either the presence (T3^+^) or absence (T3^−^) of T3 (Sigma-Aldrich, St. Louis, MO). Usually, T3 (15 nM) was applied at 0 DIV, and replenished at 7 DIV, when a third of the medium volume was refreshed. The concentration of T3 was chosen according to previously obtained dose-response curves from experiments with T3 concentrations ranging from 10^−13^ to 10^−6^ M (Westerholz et al., 2010). Significant effects were already observed at concentrations as low as 10^−12^ M. Since higher T3 concentrations yielded more consistent results without noticeable side effects, T3 was used in a concentration of 15 nM throughout this study. This is in line with other *in vitro* studies showing physiological effects of T3 at concentrations between 5 and 30 nM (Hoffmann and Dietzel, 2004; Morte et al., 2010).

### Western blotting

Protein from neocortical cultured neurons was extracted using an ice-cold RIPA lysis buffer [150 mM NaCl; 1% Igepal; 0.5% Sodium deoxycholate (Doc); 0.1% sodium dodecyl sulfate (SDS); 50 mM TrisHCl, pH 8.0] supplemented with a protease inhibitor mixture (Cømplete; Roche diagnostics GmbH, Mannheim, Germany) and phenylmethanesulfonyl fluoride (PMSF; Sigma-Aldrich). Extraction buffer was given directly to the monolayer and incubated at 4°C, over a shaker (250 rpm) for 15 min. In each experiment, samples of at least five sister cultures were pooled per age and experimental group. Debris was pelleted by centrifugation at 4°C and 13,000 rpm for 30 min. Supernatant was denaturated at 95°C for 5 min, and the protein concentration of the supernatant was determined using BCA Protein Assay Kit (Pierce by Thermo Fischer Scientific Inc., Rockford, IL). Before loading, the protein probes were diluted in Laemli sample buffer and warmed over a shaker either to 95°C for 5 min or to 37°C for 30 min, and then centrifuged (13,000 rpm) for 30 s. When samples prepared with the lower temperature method were used, fewer NKCC1 oligomeres were present in the stained blots. Samples of proteins (20–23 μg) were separated by sodium dodecyl sulfate-polyacrylamide gel electrophoresis (SDS-PAGE, either 8% or gradient gel 5–12%) and transferred onto nitrocellulose membrane (Optitran BA-S 83; Whatman, Maidstone, United Kingdom) using semi-dry method. Membranes were incubated in blocking solution (5% milk in 0.1 M PBS, 1% goat normal serum, 0.1% Tween) for at least 30 min at RT, washed once in 0.1 M PBS + 0.1% Tween (PBST) and then probed overnight at 4°C with monoclonal anti-NKCC (330 ng/ml, T4, Developmental Studies Hybridoma Bank) (Lytle et al., 1995; Zhang et al., 2006) or with polyclonal rabbit anti-KCC2 antibodies (4 μg/ml; Cat. KCC21-A; Alpha Diagnostic International Inc, San Antonio, TX) (Chee et al., 2006; Nakanishi et al., 2007) diluted in 1% milk, 1% goat normal serum, 0.1% Tween in 0.1 M PBS. Expression of either glyceraldehyde 3-phosphate dehydrogenase (GAPDH) or ß-Actin was used as a loading control (monoclonal mouse anti-GAPDH, 1:1000, clone 1D4, Covance, Princeton, NJ; monoclonal mouse Anti-β-Actin 0.5 μ l/ml, clone AC-15, Sigma). Membranes were washed thoroughly with PBST, incubated for 2 h at RT with horseradish peroxidase linked secondary antibodies: polyclonal goat anti-mouse immunoglobulins/HRP (1:10000; Cat. P0447; Dako, Glostrup, Denmark) or polyclonal goat anti-rabbit immunoglobulins/HRP (1:2000; Cat. P0448; Dako) in 0.1 M PBS, 1% milk, 1% GNS, 0.1% Tween; and washed again extensively with PBST. Detection was carried out using enhanced chemiluminescence (ECL) substrate SuperSignal West Dura (Pierce) and recorded with GeneGnome5 (Syngene by Synoptics Ldt, Cambridge, UK). The intensity of the bands was quantified using Quantity One software (Bio-Rad Laboratories Inc., Hercules, CA). Protein extracts from 6 preparations were used in three pools of up to 3 experiments in 19 blots for NKCC1 and 13 blots for KCC2. Intensity values were corrected by corresponding GAPDH or ß-actin levels. Before pooling data from different blots, the intensity values were normalized to the mean intensity of all measured bands of the same blot. Data were compared using Two-Way ANOVAs for factor hormone and factor age, followed by Holm-Sidak Method for comparisons of hormone effects within each age.

### Calcium imaging

The spontaneous network activity of cultures was recorded by using Fluo-4 Calcium Indicator (Molecular Probes, Invitrogen). Each culture was incubated with 5 μM Fluo-4 for 1 h followed by several washes with Hepes-buffered artificial cerebrospinal fluid (aCSF; in mM: 140 NaCl, 5 KCl, 1.5 CaCl_2_, 0.75 MgCl_2_, 1.25 NaH_2_PO_4_, 20 D-glucose, 15 Hepes/NaOH, pH 7.4), and 30 min at RT to allow deesterification of the dye. Sequences of fluorescence images were recorded on an inverted microscope (Axiovert, Zeiss, Oberkochen, Germany) equipped with a cooled CCD camera (CoolSNAP ES, Roper Scientific, Germany). Five fields in each culture dish were chosen randomly and activity was recorded at 1 Hz during 5-min sessions. Images were processed with MetaMorph software (vers. 7.0, Molecular Devices, Sunnyvale, CA). Fluorescence data are expressed as ΔF/F0 (background-corrected increase in fluorescence divided by resting fluorescence). The analysis of the calcium activity was done with custom-made software written in MATLAB (vs. 7.5, MathWorks, Natick, MA) and Excel (Microsoft Corp., Richmond, WA). A change in [Ca^2+^]_i_ was considered significant when the absolute difference of gray values exceeded five times the standard deviation of background noise measured in cell-free areas (Voigt et al., 2001; Opitz et al., 2002). The frequency of network bursts (activity events in which at least 10% of all cells participate), the attendance (percentage of active neurons participating in any activity event), and the single cell frequency were calculated off-line with MATLAB. Statistical tests were perfomed using SigmaStat (version 2.03, SPSS Inc., Chicago, IL).

#### GABA shift experiments

The local application of GABA agonist muscimol during calcium imaging was used to investigate the developmental changes in GABA_A_ receptor mediated synaptic transmission (Owens et al., 1996; Ganguly et al., 2001; Baltz et al., 2010). Cultures were continuously perfused with an aCSF containing the glutamate receptor antagonists 6-cyano-7-nitroquinoxaline-2,3-dione disodium (CNQX; 2.5 μM; Tocris) and D-(-)-2-amino-5-phosphonopentanoic acid (D-AP5; 12.5 μM; Tocris) to suppress spontaneous network activity. At defined time points during each recording session cells were locally perfused with an aCSF containing either high potassium (KCl, 60 mM; Merck) to identify living neurons or the GABA_A_ receptor agonist muscimol (200 μ M; Sigma-Aldrich) using a multibarreled perfusion system (Baltz et al., 2010). The local application lasted 2 s resembling prolonged neuronal activation during population burst activity. Recorded fields were marked with a diamond tool and a differential interference contrast (DIC) image of each field was made. GABAergic neurons were retrospectively identified (for identification criteria, see below) by comparing these images with corresponding images of the immunolabeled cover slips.

Images were processed off-line using MetaMorph software (Molecular Devices) and fluorescence data were processed with Excel (Microsoft Corp.). For each cell we calculated the background-corrected baseline fluorescence and the standard deviation of baseline fluctuations during a 5-s-period before local stimulant application. To detect stimulation induced changes in intracellular calcium concentration the maximal emitted fluorescence in response to local stimulation was detected. A cell was considered responsive to muscimol or KCl when the change in fluorescence exceeds five times the standard deviation of baseline fluctuations. The number of muscimol responsive cells was calculated as a fraction of KCl responsive cells per field. Five fields were probed in one culture per age (6, 9, 12, 15, 18, 21, 27 DIV) in each of three experiments. Data were compared using Two-Way ANOVAs for factor hormone and factor age, followed by Holm-Sidak Method for comparisons between ages.

#### Recovery experiments

For long-term experiments all cultures were washed twice with DMEM at 14 DIV and the medium was replaced by fresh astroglia conditioned medium supplemented either with T3 (15 nM) or without T3, resulting in four experimental groups: chronically deprived cultures (T3^−^), chronically T3-treated cultures (T3^+^), cultures treated with T3 only during the third week *in vitro* (T3^−/+^), and cultures treated with T3 during the first 2 weeks (T3^+/−^). Imaging recordings of five different fields was done in each of two cultures per experimental set (T3^−^, T3^−/+^, T3^+/−^, T3^+^), in each of two experiments.

Spontaneous calcium transients' kinetics was analyzed using Excel (Microsoft Corp., Richmond, WA) and the MiniAnalysis software (version 6.0.3, Synaptosoft, Decatur, GA). The fluorescence trace (F) of each individual cell was background corrected and normalized (ΔF/F0) according to the minimal fluorescence level during the sequence (F0). The duration of averaged calcium transients (time between the event onset and the recovery to baseline level) was calculated semiautomatically using the MiniAnalysis software (Synaptosoft). All cell recordings within a field were averaged to calculate the frequency of network activity. Differences between experimental sets were tested using Mann-Whitney Rank Sum Test (MW-RST). For comparison of multiple culturing conditions differences were tested with Kruskal-Wallis One-Way ANOVA on ranks.

#### Drug experiments

In some experiments the following drugs were added to the medium at 7 DIV and cultures were maintained until fixation time: recombinant human brain-derived neurotrophic factor (BDNF; 50 ng/ml; Pepro Tech, Rocky Hill, NJ), protein kinase inhibitor K252a (100 nM; Calbiochem by Merck KGaA, Darmstadt, Germany), or rapamycin (1.0 μM Sigma-Aldrich). K252a is a high-affinity inhibitor of the BDNF receptor tropomyosin-receptor-kinase B (trkB; Tapley et al., 1992) shown to inhibit BDNF-mediated plasticity of GABAergic neurons (Rutherford et al., 1997; Jin et al., 2003; Palizvan et al., 2004; Patz et al., 2004; Peng et al., 2010). Rapamycin is a specific inhibitor of mTOR (e.g., Swiech et al., 2008). Imaging recordings were performed in cultures of two experiments (1–2 cultures per set and experiment, five fields recorded in each culture). Data were compared using MW-RST. For comparison of multiple culturing conditions differences were tested with Kruskal-Wallis One-Way ANOVA on ranks. A possible interaction between hormone and drugs was tested with Two-Way ANOVA for factor hormone and either factor K252a or Rap.

### Immunocytochemistry

GABA-immunocytochemistry was used for identification and morphometric analysis of the neuronal populations. GABA-containing neurons were labeled with a monoclonal mouse anti-GABA antibody (Szabat et al., 1992) as described previously (de Lima and Voigt, 1997; Voigt et al., 2001; de Lima et al., 2007). In brief, cultures were fixed 30 min at 36°C by adding 70% glutaraldehyde to the culture medium to make a final concentration of 3.5%. Cultures were then thoroughly washed in Tris/metabisulfite solution (0.85% sodium metabisulfite in 0.05 M Tris buffer; pH 7.5), and preincubated for 3 h at RT (3% bovine serum albumin, 10% normal goat serum, 0.6% Triton X-100 in Tris/metabisulfite solution). After washing, cultures were incubated overnight at RT with monoclonal mouse anti-GABA antibodies (1:200; clone 5A9; Chemicon, Temecula, CA) in Tris/Metabisulfite solution containing 3% bovine serum albumin, 10% normal goat serum, 0.6% Triton X-100. After the primary antibody incubation all washes were made with phosphate buffered saline (0.1 M, pH 7.4). Primary antibodies were labeled with secondary goat anti-mouse antibody (1:200; Convance Inc, Princenton, NJ) and mouse peroxidase-anti-peroxidase (1:200, Sternberger, Baltimore, MD) in PBS containing 10% normal goat serum, 2% bovine serum albumin, 5% Sucrose and 0.3% Triton X-100 for 2 h at RT. Antibody-peroxidase complexes were made visible by 0.01% 3, 3′ diaminobenzidine tetrahydrochloride, 0.004% H_2_O_2_, 1% Nickel ammonium sulfate, 50 mM Imidazole in 50 mM Tris-HCl saline buffer. After the final PBS wash, cover slips were dehydrated in an ethanol series, cleared in two changes of xylene, and mounted over clean slides with Fluoromount (Serva, Heidelberg, Germany).

For double-immunofluorescence labeling, cover slips were fixed with 4% paraformaldehyde and 0.005% glutaraldehyde (30 min, 36°C). Anti-GABA staining was done as described above except that a rabbit anti-GABA antibody (1:20,000, generously provided by C. Beaulieu, University of Montreal) was used. After incubation with anti-GABA antibodies, cultures were washed and incubated with a secondary goat anti-rabbit Cy3 antibody (1:400, Dianova, Hamburg, Germany) for 2 h at RT. After an additional washing step, cultures were incubated with mouse anti-Synaptophysin antibodies (1:10; overnight at 4°C; Chemicon, Temecula, CA) followed by a goat anti-mouse Cy2 secondary antibody (1:400, Dianova) for 2 h at RT. After thoroughly washing cover slips were shortly dehydrated and embedded with Fluoromount. Two consecutive micrographs for the two antibody labels were taken with a CCD camera (Spot Slide) and a 100× oil immersion lens (Axiophot; Zeiss). The bandwidth of filter sets used to visualize Cy3 and Cy2 did not overlap. The pairs of micrographs were brought into register using the MetaMorph software (version 6.0; Universal Imaging Corp., West Chester, PA).

#### GABAergic neurons identification

In developing neuronal networks from dissociated E16 rat cerebral cortex two types of GABAergic neurons were described (de Lima and Voigt, 1997, 1999; Voigt et al., 2001). Small GABAergic neurons (S-GABA; Figures 4A–D, short arrows) proliferate in culture from non-GABAergic precursors after the 3rd DIV and have small fusiform to bipolar cell bodies and barely visible axons. The other GABAergic neuron type, the large GABAergic neuron (L-GABA; Figures 4A–D, long arrow), is postmitotic by the time of plating, develops a large cell body, stellate dendritic tree and form long range connections with thick, strongly stained axons. The conspicuous L-GABA has long and highly branched axons, which often surround neuronal cell bodies in a basket-like fashion, forming many boutons (de Lima and Voigt, 1997; Voigt et al., 2001; Westerholz et al., 2010). The generation of prominent GABAergic boutons is characteristic for basket subtypes of GABAergic interneurons both *in situ* (Karube et al., 2004) and in culture (Chattopadhyaya et al., 2004; Di Cristo et al., 2004). In mature cultured networks, L-GABA neurons are immunoreactive for parvalbumin, whereas S-GABA neurons are immunoreactive for calretinin (de Lima et al., 2004).

Because of their unique structure, the axons of L-GABA can easily be distinguished from dendrites, and from neurites of other GABAergic neurons, by visual evaluation of GABA-immunostained networks. Neurites of S-GABA are extremely thin and, when visible, easily differentiated from the thicker and well-stained L-GABA axons. Dendrites of L-GABA are straight, rarely branched and do not contain varicosities or boutons. In contrast, the axons of L-GABA form conspicuous nests with numerous boutons. Electron microscopic analysis had previously shown that L-GABAergic axonal varicosities (boutons), are synaptic contact sites with other neurons (Voigt et al., 2001). Double immunocytochemistry with GABA and synaptophysin antibodies showed that these boutons contain the presynaptic marker synaptophysin (Figure 5).

By changing culture protocols creating cultures with isolated GABAergic neurons (Westerholz et al., 2010) or cultures that contain exclusively one type of GABAergic interneuron, either L-GABA or S-GABA (Voigt et al., 2001), is possible. Only cultures that contain L-GABA show thicker axonal structures with many boutons (Voigt et al., 2001; Westerholz et al., 2010). Cultures containing only S-GABA show a meshwork of very fine axons, lacking thick axonal structures or conspicuous boutons (Voigt et al., 2001). This corroborates the idea that L-GABA is the source of the prominent GABAergic axons.

For simplicity, we use the term bouton for all axonal swellings visible in GABA immunostainings. That some of these boutons might not be a presynaptic structure, or that some small presynapses might remain unnoticed in these stainings, is possible. However, a misestimation should apply to experimental and control samples, and should therefore not affect the relative differences in bouton density.

#### Population analysis and morphometry

Cell density and the cell body size were quantified by focusing on the following neuronal subpopulations (de Lima and Voigt, 1997): the early born L-GABA, and the non-GABAergic neurons (non-GABA). The cell density quantification was made on an upright microscope (Standard WL; Zeiss), in 10 fields per cover slip at regularly spaced points, with the aid of a grid in the microscope eye piece. For quantification of soma area, high magnification drawings of single cell bodies were made on an upright microscope (Standard WL; Zeiss) equipped with a Camera lucida. Drawings of cell body profiles were scanned and a digitalized version was analyzed with MetaMorph software (Molecular Devices).

To quantify the number of GABAergic boutons and the local axonal length of GABAergic axons, images of randomly chosen fields were taken with a charge-coupled device camera (Spot slider, Diagnostic Instruments, Sterling Heights, MI) with a 40× objective on an upright microscope (Axiophot; Zeiss). In each image a central region of interest (120 × 90 μm) was selected and the total number of neurons and of L-GABAergic boutons was counted. Additionally, the total length of GABAergic axons was measured in each field using MetaMorph software (Molecular Devices).

Morphometric data were collected in 10 random fields of each of 2–3 cultures from each of two experiments. All quantitative data were expressed as mean ± SEM. All statistical tests were done with SigmaStat software (version 2.03, SPSS Inc., Chicago, IL). For comparison of multiple culturing conditions differences were tested with Kruskal-Wallis One-Way ANOVA on ranks (italic-underlined *P*-values shown in Tables 1–6). Two-Way ANOVA for factor hormone and factor drug treatment (BDNF, K252a or Rap) was used on ranked data to test for interactions between hormone effects and trkB or mTOR signaling pathway effects. Differences between experimental sets were additionally tested using MW-RST. Correlation between axonal parameters and local cell density was tested using Spearman Rank Order Correlation Test (SROC) or Linear Regression Test.

**Table 1:** **Summary of spontaneous activity imaging recordings of 14- and 21-day-old networks**.

		**Network frequency (bursts/min)**			**Single cell frequency (bursts/min)**			**Burst duration (s)**		
		**Mean ± s.e.m.**	***n***	***P*^#^**	**Mean ± s.e.m.**	***n***	***P*^#^**	**Mean ± s.e.m.**	***n***	***P*^#^**
14 DIV	T3^−^	5.7 ± 0.3	20		4.11 ± 0.05	1741				
	T3^+^	8.7 ± 0.5	20	<0.001	6.53 ± 0.07	1721	<0.001			
21 DIV	T3^−^	7.6 ± 0.8	20		5.54 ± 0.09	1359		7.1 ± 0.1	451	
	T3^−/+^	8.4 ± 0.5	20	0.114	7.45 ± 0.07	1360	<0.001	5.74 ± 0.05	622	<0.001
	T3^+/-^	8.3 ± 0.4	20	0.117	7.27 ± 0.08	1347	<0.001	6.14 ± 0.06	588	<0.001
	T3^+^	13.3 ± 0.5	20	<0.001	10.7 ± 0.1	1264	<0.001	3.93 ± 0.03	925	<0.001
				*<0.001*			*<0.001*			*<0.001*
				MW-RST			MW-RST			MW-RST
				*ANOVA*			*ANOVA*			*ANOVA*

Sets of cultures differed by the timing and duration of T3 treatment: T3^−^, chronically hormone-deprived; T3^+^, hormone-treated; T3^−/+^, cultures treated with T3 only during the third week in vitro; and T3^+/−^, cultures treated with T3 during the first two weeks. P^#^, P-values of T3-treated cultures compared with T3^−^. The statistical test used is indicated under the P columns. All P-values are results of Mann–Whitney Rank Sum Test (MW-RST), except the italic-underlined P-values, which are results of Kruskal–Wallis One-Way ANOVA on ranks (ANOVA).

In all tests a *P*-value of <0.05 was considered statistically significant. Asterisks in graphs show the level of statistical significance (^*^P < 0.05; ^**^P < 0.01; ^***^P < 0.001). Unless otherwise indicated, gray asterisks show the significance of the differences between drug-treated cultures and the T3-matched control. Black asterisks show the significance of the differences between T3^+^ and T3^−^ cultures in each set.

## Results

### T3 effects on NKCC1 and KCC2 expression

The depolarizing to hyperpolarizing shift of GABA contributes significantly in shaping the early synchronized activity of neocortical networks *in vitro* (Baltz et al., 2010). Here we ask if T3 has measurable effects on the GABA shift. The developmental change of GABA action is mediated by a decreased expression of the sodium-potassium-chloride cotransporter NKCC1, which accumulates chloride in young neurons, and by an increased expression of the potassium chloride cotransporter KCC2, which extrudes chloride from mature neurons (Ben-Ari et al., 2007; Blaesse et al., 2009).

**Figure 1:**
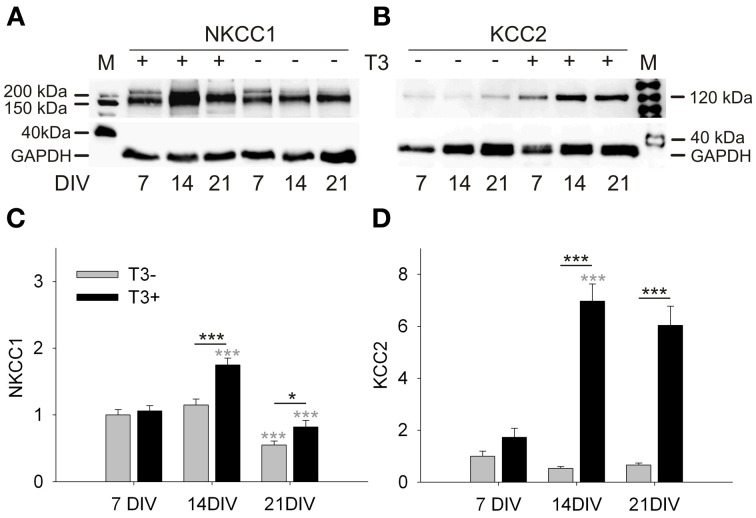
**Development of NKCC1 and KCC2 expression in T3^+^ (+) and T3^−^ (−) networks.** Representative Western blots of 7 to 21-day-old cultures stained for NKCC1 **(A)** or KCC2 **(B)**. Expression of NKCC1 and KCC2 was increased in T3^+^ cultures compared with T3^−^ cultures at 14 and 21 DIV. NKCC1 protein expression increased between 7 and 14 DIV, but decreases again until 21 DIV **(A,C)**. KCC2 protein expression did not change in function of age in T3^−^ cultures. In contrast, KCC2 expression in T3^+^ cultures increased markedly during the second week *in vitro* (**B**,**D**). Black asterisks show statistical significant differences between T3^+^ and T3^−^ networks; Gray asterisks show age-dependent differences. M, lane with molecular weight marker proteins. GAPDH protein level was used as a loading control. Values in graphs **(C,D)** were normalized to the T3^−^ value at 7 DIV.

A Western blot analysis showed that NKCC1 expression in T3^+^ cultures increased from 7 to 14 DIV, and then dropped from 14 to 21 DIV (Figures 1A,C). NKCC1 protein levels differed between T3^−^ and T3^+^ cultures at 14 and 21 DIV, but the age-dependent changes of NKCC1 expression was not influenced by the presence of T3 (*P* = 0.138). In contrast, thyroid hormone regulated the expression of KCC2 in an age-dependent manner (*P* < 0.001, Figures 1B,D). Compared with T3^−^ cultures, the expression of KCC2 was strongly increased in T3^+^ cultures at 14 and 21 DIV (*P* < 0.001, Figure 1D).

The results show that T3 specifically affected the age-dependent increase of KCC2 expression. T3 also promoted an increase of expression of NKCC1 from 7 to 14 DIV, but does not change the age-dependent decline of NKCC1 expression. The increase of KCC2 and NKCC1 expression in the presence of T3 suggests a prominent role for the hormone in chloride homeostasis and therefore also in GABAergic signaling.

### T3 modulation of the GABA shift

**Figure 2:**
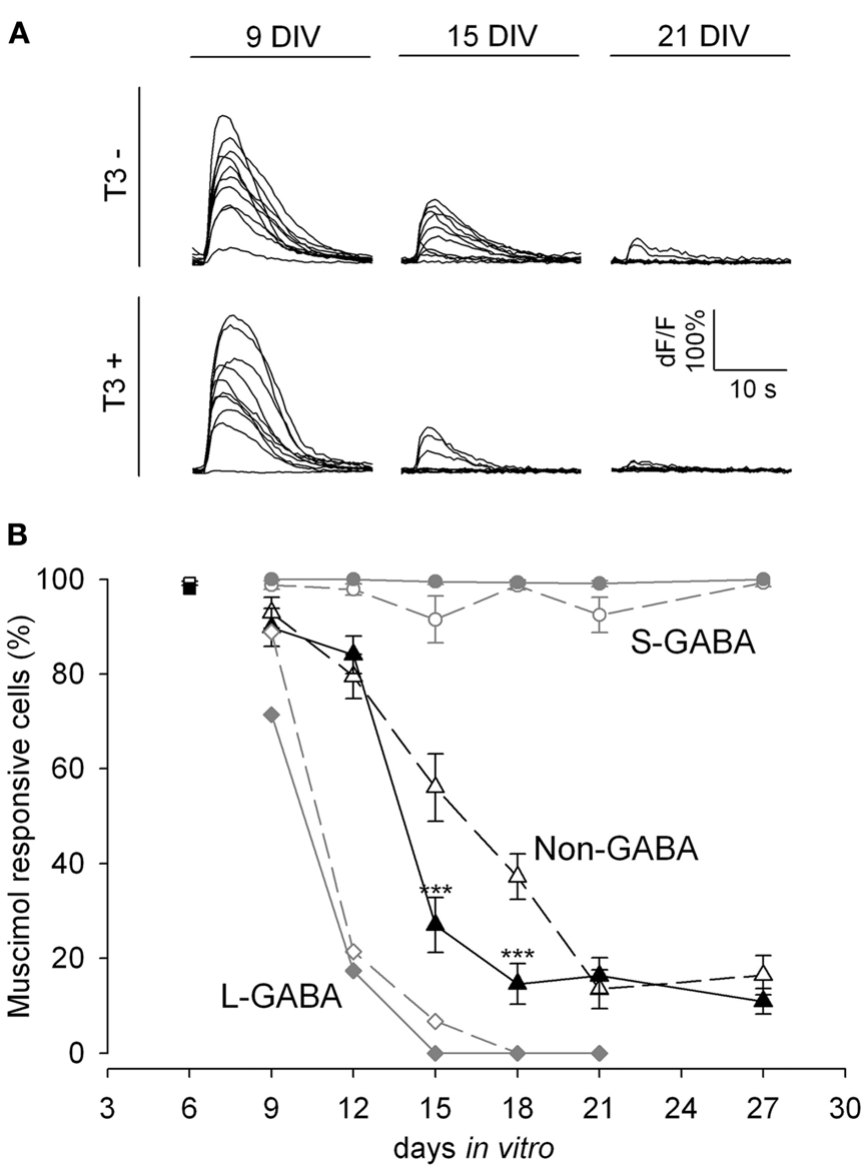
**GABA shift in T3^+^ and T3^−^ networks shown by Fluo-4 recordings. (A)**. Representative calcium transients of non-GABA grown either in T3^−^ and T3^+^ in response to muscimol application at 9, 15, and 21 DIV. **(B)**. The developmental shift from depolarizing to hyperpolarizing actions of GABA is shown by the fraction of neurons with a significant increase in fluorescence after muscimol stimulation. At 6 DIV most neurons (black and white rectangles) show calcium transients after muscimol application. Only few L-GABA (diamonds) and non-GABA (triangles) can be activated by muscimol application after day 9, but all S-GABA (circles) respond to muscimol until day 27. GABAergic neuron response to muscimol was little affected by T3 treatment. In contrast, T3 treatment accelerates the developmental downregulation of depolarizing actions of muscimol in non-GABA (black triangles).

We next tested the developmental changes in GABA_A_ receptor-mediated synaptic transmission in calcium imaging experiments by the local application of the GABA_A_ receptor agonist muscimol (Figure 2). Early bursting activity in cortical neurons is accompanied by an increase of intracellular calcium (Opitz et al., 2002). Small increases of intracellular calcium concentration are usually associated with single spiking or subthreshold activation, whereas bursting leads to a very strong increase of intracellular calcium (Opitz et al., 2002; Cossart et al., 2005). The local application of the GABA_A_R agonist muscimol early in network development evokes a calcium transient due to the depolarizing action of GABA (Yuste and Katz, 1991; Lin et al., 1994; LoTurco et al., 1995; Garaschuk et al., 2000). In this set of experiments we locally applied short puffs of either high potassium (60 mM) or muscimol (200 μ M), recorded calcium transients by Fluo-4 imaging, and counted the cells showing calcium transients. The decrease in the number of neurons showing intracellular calcium transients upon GABA_A_R activation with muscimol (Figure 2) reflects the time course of the GABA shift.

In the present experiments, an age-dependent decrease of the response to muscimol was observed (*P* < 0.001, Figure 2A). Between 6 and 12 DIV the majority of neurons (85–99%) depolarized in response to muscimol stimulation. Between 9 and 21 DIV the proportion of responsive neurons decreased markedly (*P* < 0.001). After 18 DIV less than half the neurons (33–50%) showed calcium transients after muscimol stimulation.

A separate analysis for L-GABA, S-GABA and non-GABA revealed interesting differences. Nearly all S-GABA were activated by muscimol, irrespective of age (Figure 2B, circles). In contrast, L-GABA showed an early and fast decrease in the depolarizing response to muscimol application (Figure 2B, diamonds). At 15 DIV only 3% of the L-GABA showed increased calcium influx upon muscimol stimulation. The developmental profile of non-GABA showed the expected decrease in depolarizing response to muscimol between 12 and 18 DIV (Figure 2B, triangles).

The analysis of the total neuronal population did not show any effect of T3 on the GABA shift. However, the analyses of GABAergic and non-GABAergic populations separately revealed a population-specific influence of T3 on the GABA shift of the non-GABAergic population (Figure 2B). During the transitory period, at ages 15 and 18 DIV, a sharper decrease of responsiveness to muscimol was observed in hormone-treated non-GABA. The S- and L-GABA response curves were not different among age-matched cultures in T3^−^ or T3^+^ conditions (Figure 2B).

The results show that the developmental switch of GABAergic signaling in non-GABA was accelerated in the presence of T3. The remarkable decrease in muscimol responding cells around 15 DIV was timely well-correlated to the observed changes in protein expression (see Figure 1) indicating a prominent role for KCC2 and NKCC1 in the GABA shift.

### Long-lasting changes after early T3 deficiency

The results obtained during the first 2 weeks *in vitro* (Westerholz et al., 2010) suggest that T3 influences the development of GABAergic neurons when GABA signaling is depolarizing. Because the depolarizing actions of GABAergic signaling have been implicated in the early morphological and functional maturation of neurons (Owens and Kriegstein, 2002), the developmental GABAergic shift might contribute to the closure of the T3-sensitive period of GABAergic neurons' development. If so, T3 treatment started after the GABA shift should not rescue T3-mediated alterations induced during the first 2 weeks *in vitro*. To test this hypothesis, we limited the T3 supplementation of neuronal cultures to time windows before or after the GABA shift, as determined above (see Figure 2). We set four experimental groups: chronically deprived cultures (T3^−^), chronically T3-supplemented cultures (T3^+^), cultures with T3 during the third week *in vitro* (T3^−/+^), and cultures with T3 during the first 2 weeks *in vitro* (T3^+/−^).

**Figure 3:**
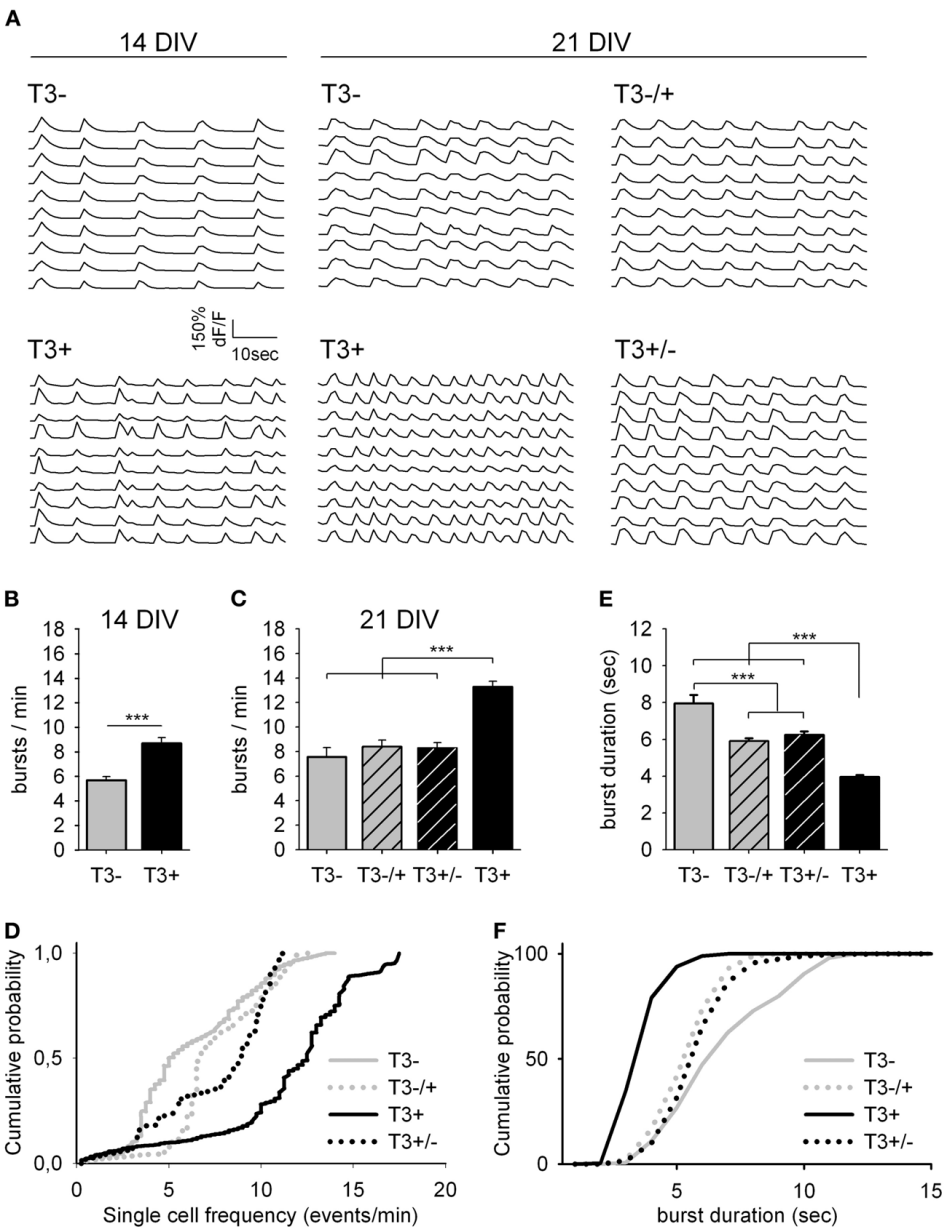
**Spontaneous network activity at 14 and 21 DIV. (A)**. Traces show the change in intracellular calcium signals of individual neurons from 14- or 21-day-old cultures grown either in the absence or presence of T3, as indicated. **(B)**. T3 increases the burst frequency at 14 DIV. **(C)**. The maximal burst frequency at 21 DIV was measured in cultures chronically treated with T3 (T3^+^). **(D)**. Graphs show the cumulative probability for single cell frequency. **(E,F)**. Burst duration at 21 DIV was reduced in T3^+^ cultures compared with T3^−^ cultures and reversal experiments. The cumulative representation of burst durations **(F)** includes all single transients of mean dF/F data (see Table 1).

**Figure 4:**
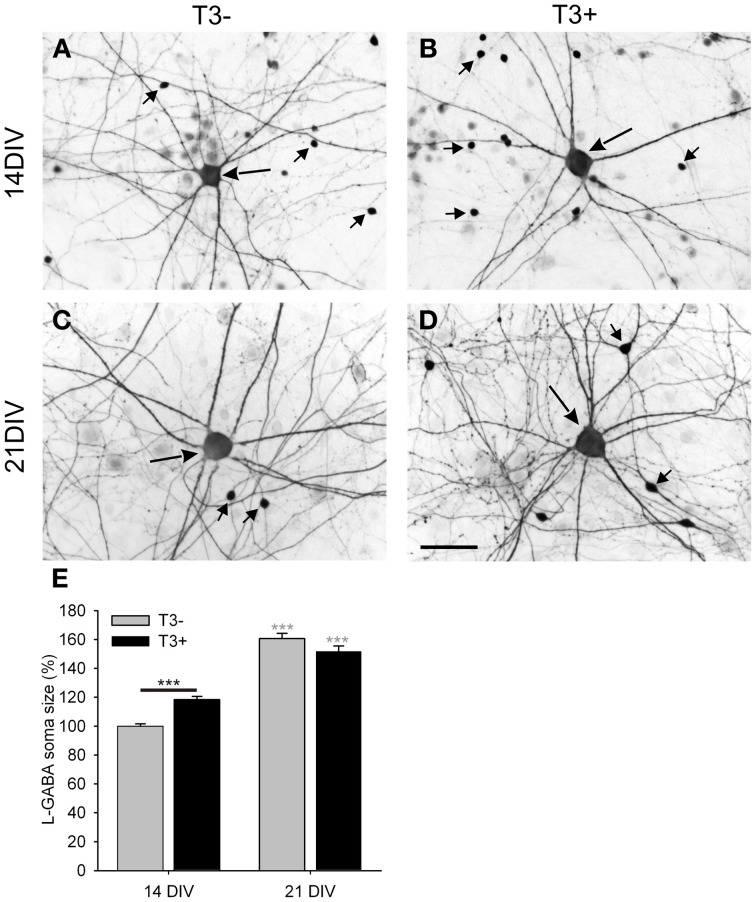
Images show GABA immunostained 14- and 21-day-old cultures grown in the absence (A,C; T3^−^) or presence (B,D; T3^+^) of T3. All images show a central L-GABAergic neuron (L-GABA, large arrow) surrounded by smaller S-GABAergic neurons (S-GABA, small arrows) and unstained non-GABAergic neurons (non-GABA). At 14 DIV L-GABA are hypertrophic in the T3^+^ cultures compared with T3^−^ cultures, but at 21 DIV L-GABA have similar soma size in T3^−^ and T3^+^ cultures. An increased number of the smaller GABAergic neurons in T3^+^ cultures are observed at 14 and 21 DIV. **(E)**. The soma size of L-GABA was not different among experimental sets at 21 DIV. Gray asterisks show the significance of the differences between 14- and 21-day-old cultures of the same treatment group. Scale bar in **(A–D)** = 50 μm.

**Figure 5:**
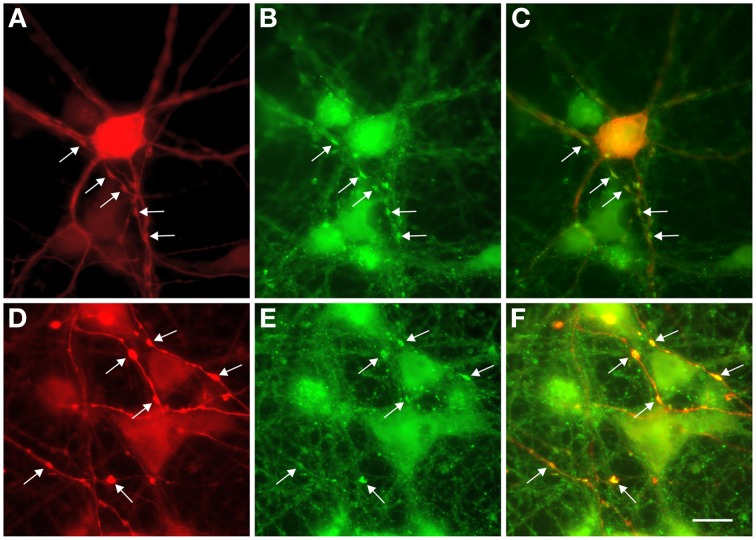
**Colocalization of GABA and synaptophysin immunostainings in 13-day-old cultures. (A,D)** GABA staining shows prominent swellings along the axons of L-GABA neurons (arrows). **(B,E)**. These boutons contain the synaptic protein synaptophysin. **(C,F)**. Double exposure of **(A,B)** and (**D,E)** shows GABA/synaptophysin colocalization. Scale bar = 10 μm.

**Figure 6:**
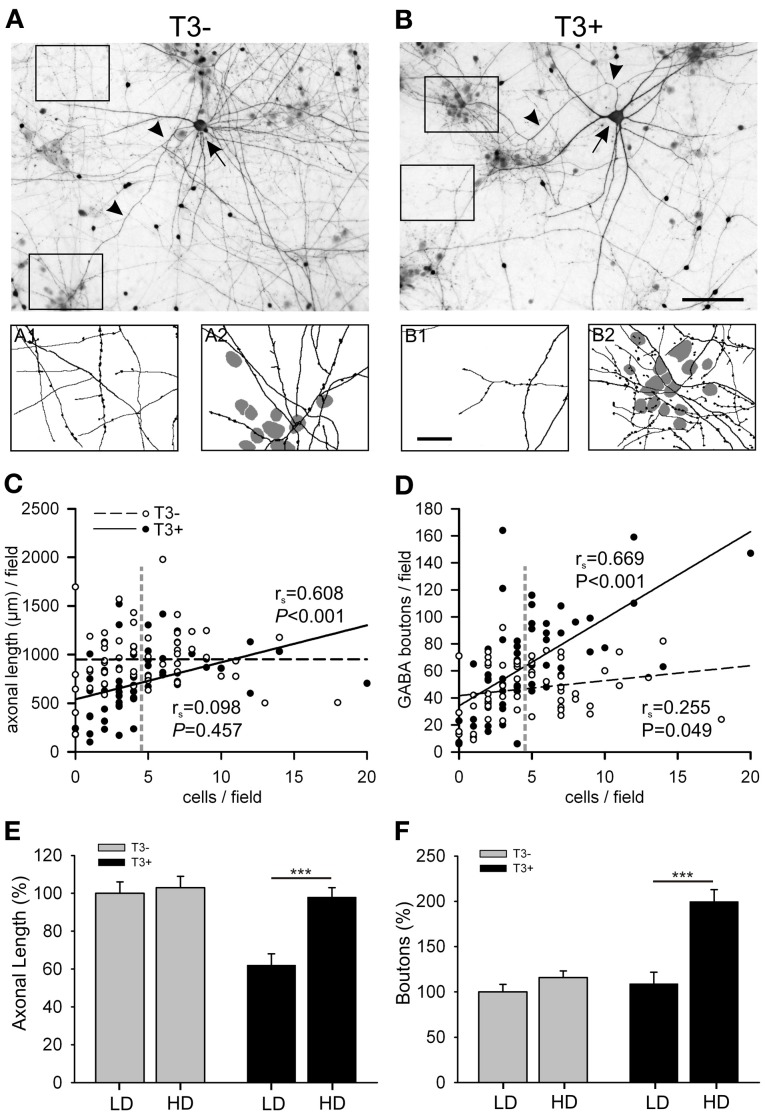
**(A,B)** Images show GABA-immunostained 14-day-old cultures grown in the absence **(A)** or presence **(B)** of T3. Each image shows a L-GABAergic neuron (arrow) with its conspicuous axons (arrow heads) surrounded by smaller S-GABA neurons and unstained non-GABAergic neurons. In T3^−^ cultures, both cell-poor (A_1_) and cell-rich (A_2_) regions are equally covered with L-GABAergic axons. In T3^+^ cultures, L-GABA axons selectively target groups of neuronal cell bodies (B_2_), but sparsely innervate cell-poor regions (B_1_). The scatter plots show that the axonal length **(C)** and the number of GABAergic boutons **(D)** are positively correlated with the local density of non-GABAergic neurons in T3^+^, but not in T3^−^ cultures. Each dot represents one field (120 × 90 μm). Lines show best linear fit. *P*-values and *r*_*s*_ coefficients are results of Spearman rank order correlation tests (SROC). Vertical dashed lines in **(C,D)** indicate the mean cell density of non-GABA neurons. This value was defined as the border between LD and HD fields (see text). **(E,F)** show means ± SEM of the data grouped according to the local cell density. LD, Low cell density fields; HD, High cell density fields; Scale bars: 100 μm in **(A,B)**; 25 μm in A_1_,A_2_ and B_1_, B_2_.

**Figure 7:**
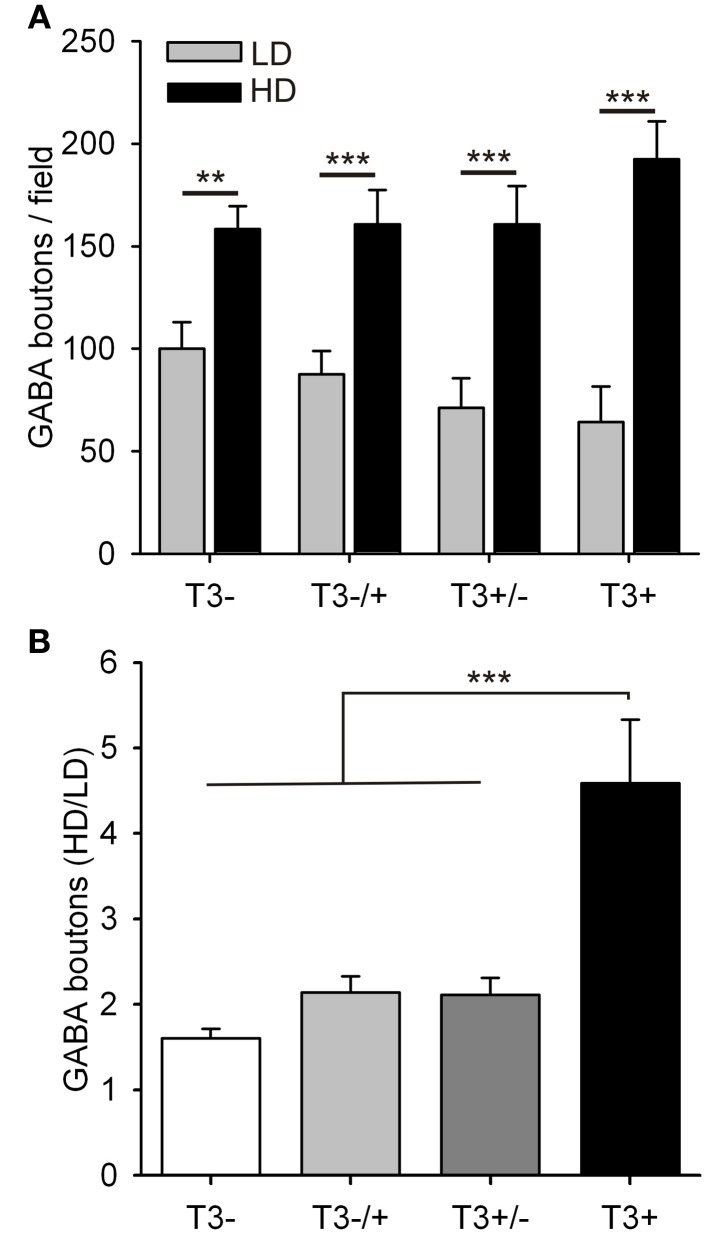
**Quantification of L-GABA axon parameters in T3^−^ and T3^+^ cultures at 21 DIV. (A,B)** Bouton density data were grouped into fields, with low (≤3 non-GABA/field) or high (>3 non-GABA/field) cell densities. **(A)**. In all culture conditions the number of GABAergic boutons was increased in fields with high neuronal density compared with fields of low neuronal density. **(B)**. The ratios of bouton numbers (high/low density fields, HD/LD) did not recover when neurons were treated with T3, after a T3 deficit during the first 2 weeks *in vitro* (T3^−/+^).

We first examined the patterns of spontaneous network activity with calcium imaging (Figure 3, Table 1). Additionally, we examined neuronal populations and GABAergic morphological development after GABA immunocytochemistry. Three aspects of the neuronal growth are considered here: the development of the L-GABAergic and non-GABA populations (Figure 4, Table 2), the development of the soma size (Figure 4, Table 2), and axonal growth of early born L-GABA, including axonal extension, and bouton formation (Figures 5–7, Table 3).

**Table 2:** **Summary of L-GABA population density and soma size**.

		**L-GABA**						**Non-GABAergic**		
		**Population (cells/mm^2^)**			**Soma (size μm^2^)**			**Population (cells/mm^2^)**		
		**Mean ± s.e.m.**	***n***	***P*^#^**	**Mean ± s.e.m.**	***n***	***P*^#^**	**Mean ± s.e.m.**	***n***	***P*^#^**
14 DIV	T3^−^	7.3 ± 0.5	60		304 ± 5	120		667 ± 35	60	
	T3^+^	6.2 ± 0.6	60	0.070	359 ± 6	120	<0.001	464 ± 27	60	<0.001
21 DIV	T3^−^	4.6 ± 0.4	60		485 ± 10	120		410 ± 18	60	
	T3^−/+^	4.4 ± 0.4	60	0.909	471 ± 11	120	0.216	370 ± 16	60	0.127
	T3^+/−^	3.8 ± 0.3	60	0.290	473 ± 10	119	0.351	370 ± 19	60	0.095
	T3^+^	3.6 ± 0.4	60	0.067	463 ± 10	120	0.092	385 ± 16	60	0.259
				*0.196*			*0.401*			*0.305*
				MW-RST			MW-RST			MW-RST
				*ANOVA*			*ANOVA*			*ANOVA*

Population statistics of non-GABA is also shown. Treatment groups and statistics as in Table 1. n, number of fields (L-GABA and non-GABA populations) or number of L-GABA (soma size).

**Table 3:** **Summary of L-GABAergic bouton counts**.

		**GABA boutons**							
		**Counts/Field**					**Counts HD/LD**		
		**Mean ± s.e.m.**	***n***	***P^#^***	**r_s_**	***P*^s^**	**Mean ± s.e.m.**	***n***	***P^#^***
14 DIV	T3^−^	47 ± 3	60		0.255	0.049			
	T3^+^	64 ± 5	60	0.009	0.669	<0.001			
21DIV	T3^−^	91 ± 6	40		0.543	<0.001	1.6 ± 0.1	23	
	T3^−/+^	84 ± 7	40	0.368	0.647	<0.001	2.1 ± 0.2	21	0.018^*^
	T3^+/−^	76 ± 8	40	0.086	0.761	<0.001	2.1 ± 0.2	18	0.072
	T3^+^	76 ± 10	40	0.074	0.855	<0.001	4.6 ± 0.7	18	<0.001
				0.*512*					<*0.001*
				MW-RST	SROC				MW-RST
				*ANOVA*					*ANOVA*

Treatment groups and statistics as in Table 1. Additionally r_s_ and P^s^ are results of Spearman rank order correlation tests (SROC). To obtain HD/LD ratios, the bouton counts in HD fields were divided by the mean of LD fields in each experiment. n, number of fields examined.(

* not significant, P > 0.05, according to KW-ANOVA on ranks, followed by multiple comparisons using Dunn's method).

#### Spontaneous network activity

T3 modulates the excitability level of the network, and these changes of spontaneous activity mediate T3 actions on GABAergic neurons' morphological development (Westerholz et al., 2010). Spontaneous activity during the early network development is characteristically confined to bursts of action potentials, which is reflected in an increased intracellular calcium concentration ([Ca^2+^]_i_) (Opitz et al., 2002). Early network oscillations are expressed between the first appearance of activity bursts synchronized among a small number of neurons, and the disappearance of the calcium signal (*in vitro* during the fourth week; *in vivo* during the second week postnatal). During this period, long, robust and widely synchronized network bursts play a role in the circuitry formation (Katz and Shatz, 1996; Garaschuk et al., 2000; Voigt et al., 2005).

Here, the functional development of neuronal networks supplemented with T3 during different time windows was estimated using the calcium imaging technique (Figure 3). All 14- and 21-day-old networks showed prominent synchronous activity events (Figure 3A). The supplementation of the medium with T3 enhanced burst frequency at 14 DIV and at 21 DIV (Figures 3B,C, Table 1). An early onset and continuity of T3 supplementation were necessary for the increase of burst frequency, and a late supplementation did not rescue the decreased burst frequency due to previous hormone deprivation.

The analyses of single cell activity also showed that at 14 and 21 DIV, neurons in T3^+^ networks showed higher single cell burst frequencies than neurons in T3^−^ networks (Figure 3D, Table 1). Neurons in networks with a late addition or late deficit of T3 had intermediate burst frequencies, higher than T3^−^, but lower than T3^+^. Both the number of active neurons and the relative participation in single bursts did not vary between 21-day-old T3^+^ and T3^−^ networks.

As a general indicator of burst kinetics, the duration of calcium transients was estimated in 21-day-old cultures (Figures 3E,F, Table 1). The longest calcium transients were measured in T3^−^ networks and the shortest in T3^+^ networks. Calcium transients showed intermediate durations when T3 treatment was limited to the first 2 weeks or to the third week *in vitro*. These results show that a constant supply of T3 is correlated with an increased burst frequency and a decrease of burst duration. Hormone addition after the initial period of development (i.e., at the third week *in vitro*) does not completely rescue deficits associated with the early absence of T3.

#### Neuronal populations

Here we analyzed the development of GABAergic and non-GABAergic neuronal densities at 14 and 21 DIV (Table 2). According to earlier results (Westerholz et al., 2010), the L-GABA density was not changed by hormone treatment. Also at 21 DIV, the L-GABA density did not vary according to T3 treatment. As a control the density of non-GABA was also analyzed. The presence of T3 translates in fewer neurons in the network (Table 2), an effect possibly correlated with the increased network activity (Figure 3) and the onset of natural cell death. At 14 DIV, non-GABA density decreased in T3^+^ cultures compared with T3^−^ cultures. At 21 DIV, however, the neuronal density in T3^−^ and T3^+^ cultures equalized (Table 2), probably due to a delayed decrease in neuronal density in T3^−^ cultures. The overall reduction in non-GABA density may follow the activity-dependent intrinsic cell death program, and is in line with a generally accelerated maturation by T3.

#### Soma size

Although L-GABA showed a prominent T3-mediated enlargement of soma size in 14-day-old networks, in 21-day-old networks L-GABA soma size was similar in all experimental sets (Figures 4A–D, Table 2). Apparently, L-GABA (Figure 4E), as also S-GABA and non-GABA (data not shown), grew prominently between 14 and 21 DIV, so that earlier differences, due to different hormone treatments, disappeared.

#### Early axonal growth

In cultured networks, the formation of synapses is promoted in the presence of T3 (Westerholz et al., 2010). To analyze in detail the influence of T3 on the axonal maturation of early born L-GABA, the total length of their characteristically thick axons was measured in randomly defined fields. Additionally, boutons were counted as putative synaptic contact sites in each field (Figure 5; Voigt et al., 2001).

Irrespective of the presence or absence of T3, non-GABA neurons often clustered into small groups and form cell-rich and cell-poor areas (Figures 6A,B, squares). In contrast, the axons of GABAergic and non-GABA grew to form a continuous network. In T3^+^ cultures, L-GABAergic axons and boutons concentrate around the non-GABA soma clusters (Figure 6B), whereas L-GABAergic axons in T3^−^ cultures distributed more homogeneously throughout the neuronal network (Figure 6A). This is relevant because the morphological and immunocytochemical features of L-GABA neurons strongly indicate that they develop in a basket-type interneuron, forming many synapses near or at the cell body of non-GABA postsynaptic neurons (Somogyi, 1989; de Lima and Voigt, 1997; Tamas et al., 2000; Voigt et al., 2001).

To quantify this observation, the total number of neurons was counted in each field and we tested if the T3 modulation of axonal parameters varies according to the local non-GABA cell body density. The total axonal length and the number of boutons were positively correlated with the local cell density in T3^+^ cultures (Figures 6C,D, solid lines). In T3^−^ cultures the axonal parameters were not correlated with the local density of non-GABAergic cell bodies (Figures 6C,D, dashed lines). The correlation analysis suggests that T3 treatment favored GABAergic bouton formation near neuronal somata, and selectively reduced axonal extension in fields with few or no neuronal cell bodies. The data was then grouped according to the local cell density: fields with non-GABA counts lower or higher than the mean non-GABA counts/field in control cultures were designated to categories “low cell density” (LD) or “high cell density” (HD), respectively (Figures 6E,F). The Two-Way ANOVA analyses of the data confirmed that T3 effects on axonal growth depend on local circuitry (Length, *P* = 0.005, Boutons, *P* = 0.001).

#### Late axonal growth

For further analysis of the long-lasting effects of T3, the influence of the local circuitry was taken in consideration. We focused at the hormone effect on the number of boutons formed by L-GABA.

The data was again grouped according to the local cell density (Figure 7). Although the total number of GABAergic boutons formed by L-GABA did not differ between T3^+^ and T3^−^ cultures at 21 DIV (Table 3), the grouped data showed that the HD/LD ratio was larger in T3^+^ cultures compared with other experimental sets (Figure 7B, Table 3). Thus, T3 selectively promoted the growth of L-GABA boutons around non-GABA cell bodies during the first 2 weeks of development *in vitro*. The addition of T3 after this period did not reverse deficits of a previous deprivation.

### Contribution of trKB and mTOR signaling pathways to T3-dependent development

The neurotrophin BDNF is well-known as a mediator of activity-dependent effects and is an important regulator of interneuron development (Palizvan et al., 2004; Patz et al., 2004; Woo and Lu, 2006; Huang et al., 2007; Huang, 2009). Previous results showed complementary effects of T3 and neuronal activity in the regulation of L-GABA development (Westerholz et al., 2010) and T3 has been suggested to regulate BDNF expression (Koibuchi et al., 1999; Koibuchi and Chin, 2000). Another possible effector of T3 effects is mTOR. The serin/threonine protein kinase mTOR has been shown to both mediate thyroid hormone effects and to influence the interneuron development (Moeller et al., 2006; Sui et al., 2008; Fu et al., 2012).

**Figure 8:**
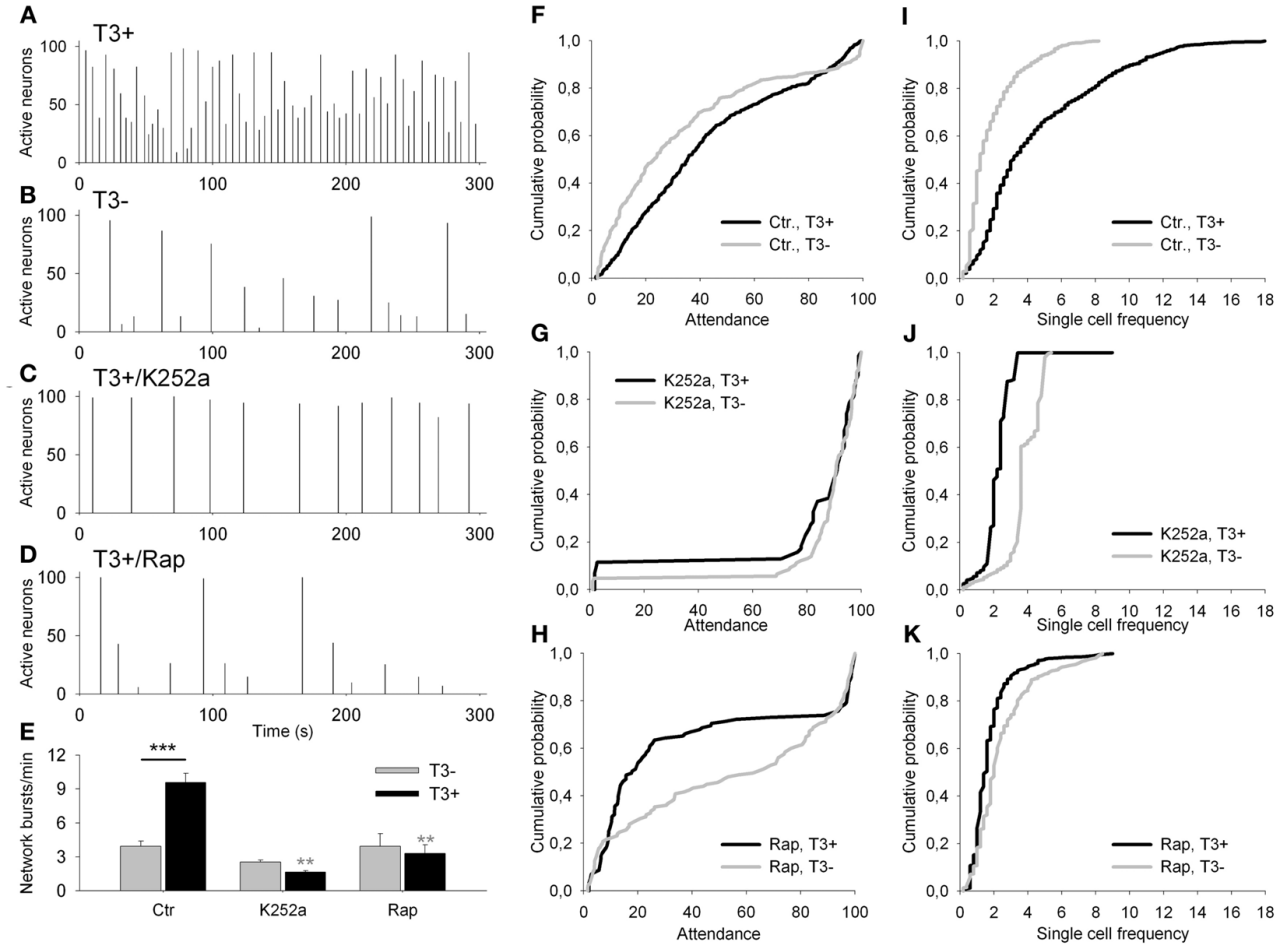
**(A–D)** Network activity histograms of representative fields recorded at 13–14 DIV with Fluo-4 calcium imaging. Bars show the percentage of active neurons against time. **(E)** The graph shows the network burst frequency for T3^−^ (gray bars) and T3^+^ (black bars) in control sets of cultures (Ctr), and in cultures treated chronically with K252a or rapamycin (Rap). **(F–K)** Graphs show the cumulative probability for attendance (% of active cells participating in single activity events) **(F–H)** and single cell frequency **(I–K)** for each of control **(F,I)** and drug-treated culture sets (K252a: **G,J**; rapamycin: **H,K**).

**Figure 9:**
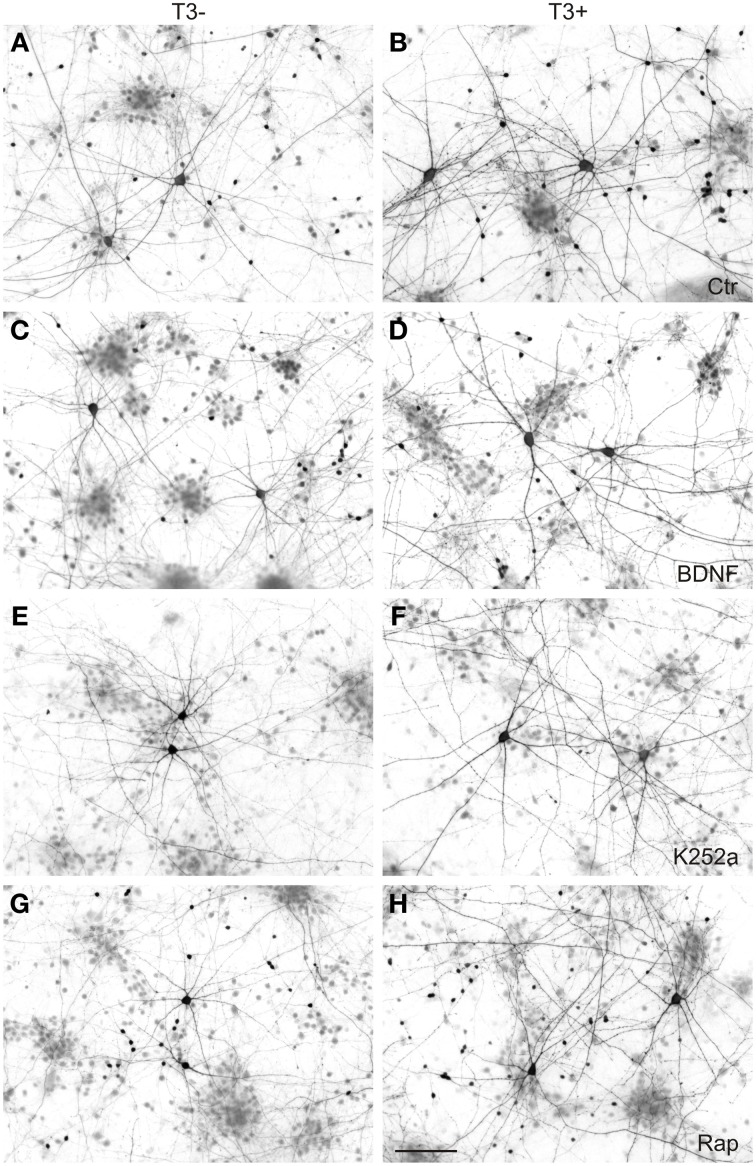
**GABAergic neurons in 14-day-old neocortical networks grown with or without T3.** GABA immunocytochemistry; T3^−^, left; T3^+^, right; Ctr, control cultures **(A,B)**; BDNF **(C,D)**; K252a, cultures treated with the antagonist to block trkB pathway **(E,F)**; Rap, cultures treated with the mTOR antagonist rapamycin **(G,H)**. Scale bar = 100 μm.

**Figure 10:**
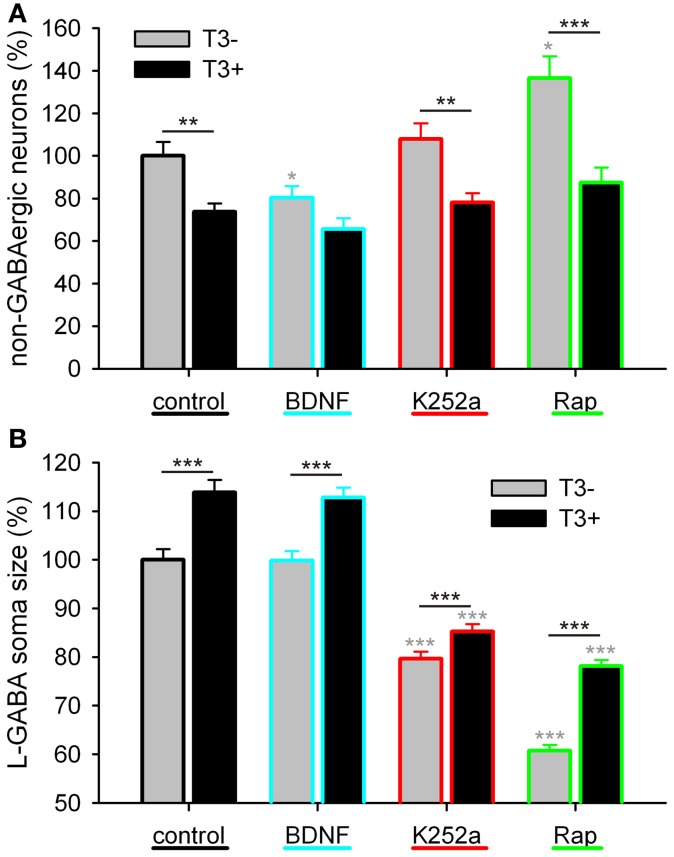
**Effect of trkB or mTOR signaling pathways on the T3-dependent development of non-GABA population (A) and GABAergic soma growth (B).** Note that, on the non-GABA population, the T3 influence decreases in the presence of BDNF, and apparently increases with the blockade of mTOR by rapamycin **(A)**. The L-GABA population showed decreased soma sizes when growing without either T3, trkB or mTOR signaling **(B)**. Data normalized to the T3^−^ control value.

**Figure 11:**
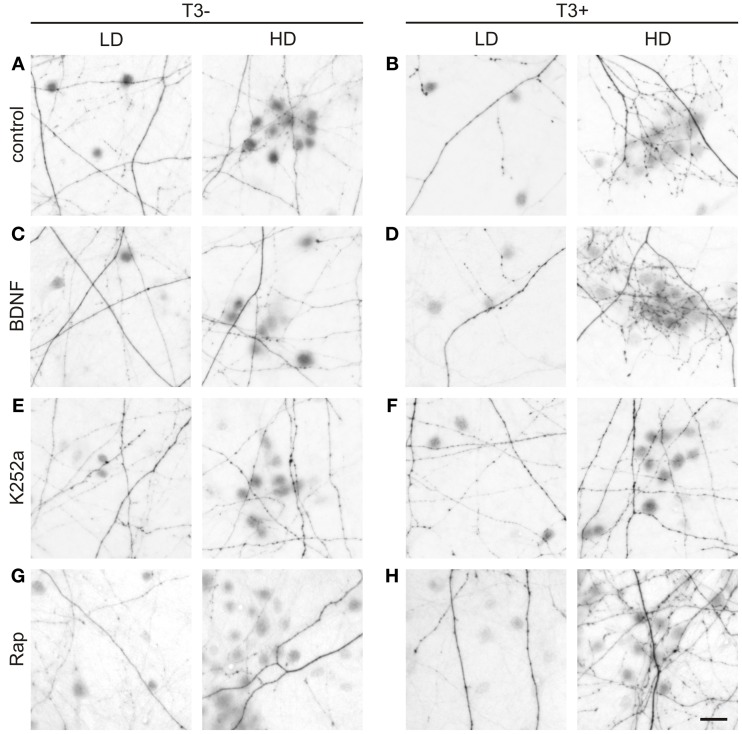
**T3-dependent distribution of L-GABA axons and boutons and the effect of trkB and mTOR signaling.** Images show GABAergic innervation patterns of LD or HD areas in 14-day-old T3^−^ **(A)** and T3^+^ control cultures **(B)**, T3^−^ and T3^+^ cultures treated with BDNF **(C,D)**, T3^−^ and T3^+^ cultures treated with K252a **(E,F)**, and T3^−^ and T3^+^ cultures treated with rapamycin **(G,H)**. Scale bar = 20 μm.

**Figure 12:**
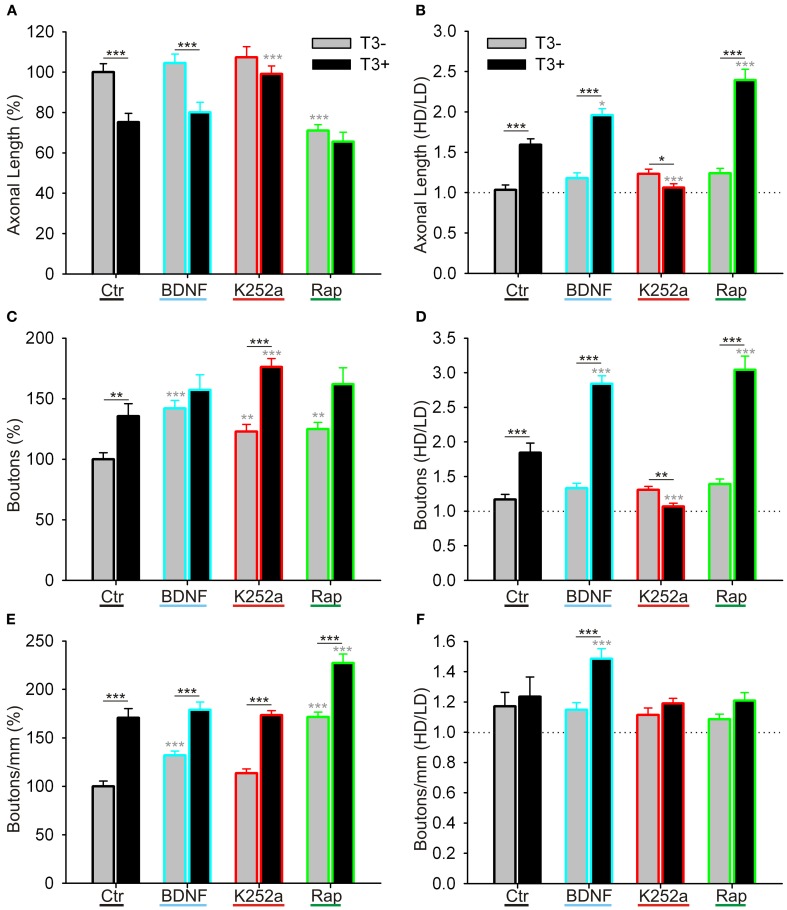
**Quantitative measurements of L-GABAergic axons and boutons.** Graphs illustrate the effect of T3 (gray bars: T3^−^; black bars: T3^+^), of BDNF (blue contours), trkB blockade (red contours), and mTOR blockade (green contours) on GABAergic axonal length/field **(A,B)**, bouton density/field **(C,D)**, and bouton density/mm axon **(E,F)**. **(A,C,E)** show means ± SEM of all fields, normalized to T3^−^ control; **(B,D,F)** show the ratio of the data grouped by the local cell density (see text for details). LD, Low cell density fields; HD, High cell density fields; Ctr, Control networks; K252a, blocker of trkB signaling; Rap, rapamycin, blocker of mTOR signaling.

To investigate the interplay between BDNF signaling and T3-mediated growth of L-GABA we added either exogenous BDNF (50 ng/ml) or the tyrosine kinase inhibitor K252a (100 nM) to the cultures, alone or in combination with T3. K252a is a high-affinity inhibitor of the BDNF receptor trkB (tropomyosin-related kinase B; Tapley et al., 1992) shown to inhibit BDNF-mediated plasticity of GABAergic neurons (Rutherford et al., 1997; Jin et al., 2003; Palizvan et al., 2004; Patz et al., 2004; Peng et al., 2010). mTOR signaling effect was probed with the antagonist rapamycin (1.0 μM). Rapamycin is a naturally occurring antibiotic that acts as a specific, allosteric inhibitor of mTORC1 (Dowling et al., 2010). Also mTORC2, thought to be resistant to rapamycin, shows sensitivity to prolonged treatment, which interferes with de novo assembly of mTORC2 (Dowling et al., 2010). In the following experiments, we tested if BDNF or mTOR acts as an effector of T3 actions on network activity and early GABAergic neurons growth during neocortical network development (Figures 8–12, Tables 4–6).

**Table 4:** **Summary and statistical analysis of spontaneous activity imaging recordings of 14 day-old networks and T3, K252a, and rapamycin effects**.

	**T3^−^**			**T3^+^**			**T3^−^ × T3^+^**	**Drug × T3**
	**Mean ± s.e.m.**	***n***	***P*^#^**	**Mean ± s.e.m.**	***n***	***P*^#^**	P^*^	***P*^&^**
**NETWORK FREQUENCY (bursts/min)**								
Control	3.9 ± 0.4	10		9.6 ± 0.8	10		<0.001	
K252a	3.3 ± 0.8	5	0.501	1.6 ± 0.1	5	0.003	0.095	<0.001
Rapamycin	4 ± 1	5	0.426	2.5 ± 0.2	5	0.003	0.548	<0.001
			*0.614*			*<0.001*		
**ATTENDANCE (%neurons/event)**								
Control	34 ± 2	276		42 ± 1	576		<0.001	
K252a	70 ± 4	83	<0.001	75 ± 5	49	<0.001	0.985	0.412
Rapamycin	54 ± 3	125	<0.001	38 ± 4	115	0.005	0.037	<0.001
			*<0.001*			*<0.001*		
**SINGLE CELL FREQUENCY (bursts/min)**								
Control	1.85 ± 0.05	799		4.6 ± 0.1	880		<0.001	
K252a	2.33 ± 0.02	632	<0.001	1.54 ± 0.02	449	<0.001	<0.001	<0.001
Rapamycin	2.47 ± 0.08	455	<0.001	1.78 ± 0.06	439	<0.001	<0.001	<0.001
			*<0.001*			*<0.001*		
			MW-RST			MW-RST		2w-ANOVA
			*ANOVA*			*ANOVA*		

P^#^, P-value of drug treatment groups vs. untreated controls of the same hormone category; P^*^, P-value of T3^+^ vs. T3^−^ of the same drug category. P^&^, P-values of the interaction between T3 and drug effects. The statistical test used is indicated under the P columns. P^#^ and P^*^-values from Mann–Whitney Rank sum test (MW-RST), except the italic-underlined P-values, which are results of Kruskal–Wallis One-Way ANOVA on ranks (ANOVA). P^&^, P-values obtained with Two-Way ANOVA. n, number of fields (Network frequency), number of events (Attendance), and number of cells (Single cell frequency) analyzed per condition.

#### Network activity

First, we asked whether the blockade of trkB or mTOR signaling interferes with the T3-dependent development of network activity. Acute treatment with either K252a or rapamycin at 7–8 DIV, time of onset of chronic treatment, results in a marked decrease in network burst frequency, attendance in single events and single cell excitability compared with control recordings (data not shown). The quantitative results obtained after chronic drug treatment are summarized in Figure 8 and Table 4. The chronic drug treatment with K252a or rapamycin did not alter significantly the mean number of active neurons or the maximal burst attendance registered in each field (Two-Way-ANOVA, *P* = 0.082 and *P* = 0.046, respectively).

Networks grown with T3 in the culture medium showed higher network burst frequency (Figures 8A,B,E, Table 4), with more active neurons participating in single network events (Figure 8F) and more cells showing higher bursting frequencies (Figure 8I) compared with networks lacking the hormone.

After the 6-day treatment with K252a (Figures 8C,E,G,J, Table 4), network burst frequency decreased (Figure 8E), and single cell frequency declined compared with controls (Figure 8J). Most network bursts showed more than 80% of neurons participating, resulting in an increased network burst attendance (T3^+^/Control, median = 35.35%; T3^+^/K252a, median = 93.67%; Figure 8G). These results indicate a decline of excitability and an increase of network synchronization after the blockade of trkB signaling.

The chronic blockade of mTOR signaling with rapamycin decreased both the network burst frequency and the frequency of single cells (Figures 8D,E,K, Table 4). Most events had very low (<30%) or very high (>80%) attendances, resulting in the decline of overall attendance and the virtual disappearance of events with intermediate attendance (T3^+^/Control, median = 35.35%; T3^+^/Rap, median = 17.95%; Figure 8H). Thus, when treated chronically with rapamycin, network excitability decreased with a concomitant decrease of relative network synchronization.

T3 influence on network and single cell bursting frequencies was modified by the blockade of both trkB and mTOR signaling pathways (Table 4). Burst synchronization was affected by trkB and mTOR signaling, but significant modification of T3-dependent changes in network synchronization was detected only after mTOR signaling blockade (Table 4).

#### Neuronal populations

In previous results (Table 2; Westerholz et al., 2010), the density of L-GABA was not affected by hormone treatment (Figure 9, Table 5). Similarly, alterations of trkB or mTOR signaling showed no effect in the number of L-GABA present in the networks (Table 5).

**Table 5:** **Summary of neuronal population quantification, L-GABAergic cell size measurements, and of the GABAergic axon parameters**.

	**T3^−^**			**T3^+^**			**T3^−^**× **T3^+^**	**Drug × T3**
	**Mean ± s.e.m.**	***n***	***P*^#^**	**Mean ± s.e.m.**	***n***	***P*^#^**	P*	***P*^&^**
**L-GABA (cells/mm^2^)**								
Control	4.4 ± 0.4	60		4.4 ± 0.3	60		0.937	
BDNF	4.7 ± 0.4	60	0.643	5 ± 1	50	0.253	0.138	0.255
K252a	5.1 ± 0.5	60	0.603	5.1 ± 0.5	60	0.338	0.739	0.806
Rapamycin	5.6 ± 0.4	60	0.070	4.4 ± 0.5	50	0.803	0.078	0.163
			*0.310*			*0.308*		
**non-GABA (cells/mm^2^**)								
Control	708 ± 46	60		521 ± 29	60		0.003	
BDNF	569 ± 38	60	0.029	465 ± 36	50	0.129	0.074	0.543
K252a	763 ± 53	60	0.605	553 ± 31	60	0.537	0.004	0.939
Rapamycin	967 ± 72	60	0.007	618 ± 51	50	0.326	<0.001	0.398
			<*0.001*			*0.095*		
**GABA SOMA SIZE (μm^2^**)								
Control	308 ± 7	120		351 ± 8	120		<0.001	
BDNF	308 ± 6	120	0.835	348 ± 6	120	0.572	<0.001	0.825
K252a	246 ± 4	120	<0.001	264 ± 5	120	<0.001	0.004	0.300
Rapamycin	187 ± 4	120	<0.001	241 ± 4	120	<0.001	<0.001	<0.001
			<*0.001*			<*0.001*		
**GABA AXON LENGTH (μm/field)**								
Control	951 ± 40	60		715 ± 42	60		<0.001	
BDNF	994 ± 42	60	0.657	762 ± 47	60	0.487	0.001	0.703
K252a	1020 ± 50	60	0.300	943 ± 38	60	<0.001	0.397	0.027
Rapamycin	676 ± 28	60	<0.001	624 ± 44	60	0.160	0.312	0.009
			<*0.001*			<*0.001*		
**GABA BOUTONS (counts/field)**								
Control	47 ± 3	60		64 ± 5	60		0.009	
BDNF	67 ± 3	60	<0.001	74 ± 6	60	0.309	0.836	0.038
K252a	58 ± 3	60	0.009	83 ± 3	60	<0.001	<0.001	0.174
Rapamycin	58 ± 3	60	0.003	76 ± 6	60	0.282	0.200	0.244
			<*0.001*			*0.023*		
			MW-RST			MW-RST		2w-ANOVA
			*ANOVA*			*ANOVA*	

Statistics as in Table 4. n, number of fields analyzed per condition.

As in the other experiments (see Table 2; Westerholz et al., 2010), T3-treated networks showed fewer non-GABA neurons (Figure 10A, Table 5). BDNF decreased non-GABAergic population only in T3^−^ set, abolishing the difference between T3^−^ and T3^+^ sets. The density of non-GABA in K252a-treated cultures did not differ from control cultures. In contrast, rapamycin increased non-GABA population in T3^−^ cultures, increasing the difference between T3^−^ and T3^+^ sets. Thus, in the absence of T3, BDNF and rapamycin may influence, in opposite ways, the development of the non-GABA population. However, an interaction of either BDNF or rapamycin with T3 effects was not corroborated by the Two-Way ANOVA (Table 5, P^&^).

#### L-GABAergic soma size

The soma size of L-GABA was measured in T3-treated and T3-untreated cultures after application of BDNF, K252a, or rapamycin. As observed earlier (Table 2; Westerholz et al., 2010), at 14 DIV, the soma size of L-GABA differed consistently between T3^−^ and T3^+^ cultures (Figures 9A,B, Table 5). The increase in cell body size in T3^+^ cultures, compared with T3^−^ cultures, was observed in both control and drug-treated cultures (Figure 10B). The comparison of drug-treated cultures with control cultures inside each T3 treatment group showed that BDNF had no effect (Figures 9C,D, 10B, Table 5), but both K252a and rapamycin decreased L-GABA soma growth (Figures 9E–H, 10B, Table 5). Interestingly, rapamycin treatment increased the difference between L-GABA soma size in T3^−^ and T3^+^ cultures (Figure 10B). This suggests that the T3 effect on L-GABA growth may vary according to the activation status of the m-TOR complex (Table 5, P^&^).

#### GABAergic axons length

Next we measured axonal length in control and drug-treated cultures in both T3^−^ and T3^+^ conditions (Figures 11, 12, Table 5). In control cultures, the axonal parameters were modulated by T3 treatment: axonal length decreased in T3^+^ cultures (Figure 12A, Table 5). The difference in axonal extension between T3^−^ and T3^+^ remained unchanged after treatment with BDNF, but disappeared after treatment with K252a or rapamycin. In the T3^−^/K252a cultures, axonal length did not differ from control, whereas T3^+^/K252a cultures showed increased axonal length. Conversely, in T3^−^/Rap cultures axonal length was decreased compared with T3^−^/control, whereas in T3^+^/Rap cultures axonal length did not differ from control.

Then, because the sampled fields differed in the local density of neuronal cell bodies, the influence of the local circuitry was taken in consideration in the analysis of axonal parameters. The data was grouped according to the number of non-GABA somata (see Figure 6): fields with less or more cells than the mean density of all fields in control cultures (4.85 ± 0.48 cells/field in T3^−^ and 4.60 ± 0.47 cells/field in T3^+^) were designated to categories LD and HD, respectively (Figures 11, 12, Table 6).

**Table 6:** **Summary of the axonal parameters considering circuitry context**.

	**T3^−^**			**LD × HD**	**T3^+^**			**LD × HD**	**T3^−^ × T3^+^**
	**Mean ± sem**	***n***	***P*^#^**	***P*°**	**Mean ± sem**	***n***	***P*^#^**	***P*°**	P^*^
**AXON LENGTH**									
**LD fields (μm/field)**									
Control	938 ± 54	33			577 ± 53	36			<0.001
BDNF	935 ± 52	38	0.734		561 ± 46	37	0.812		<0.001
K252a	871 ± 120	15	0.306		923 ± 126	7	0.022		0.573
Rapamycin	589 ± 41	23	<0.001		340 ± 43	24	0.002		<0.001
			<*0.001*				<*0.001*		
**HD fields (μm/field)**									
Control	966 ± 59	27		0.941	921 ± 43	24		<0.001	0.671
BDNF	1094 ± 69	22	0.181	0.071	1084 ± 45	23	0.012	<0.001	0.829
K252a	1070 ± 52	45	0.143	0.052	945 ± 40	53	0.506	0.800	0.125
Rapamycin	729 ± 35	37	0.001	0.064	813 ± 45	36	0.108	<0.001	0.129
			<*0.001*				*0.003*		
**GABA BOUTONS**									
**LD fields (counts/field)**									
Control	44 ± 4	33			48 ± 6	36			0.843
BDNF	60 ± 4	38	0.014		43 ± 4	37	0.808		0.004
K252a	47 ± 6	15	0.815		78 ± 9	7	0.009		0.020
Rapamycin	48 ± 4	23	0.351		35 ± 4	24	0.141		0.015
			*0.036*				*0.006*		
**HD fields (counts/field)**									
Control	51 ± 3	27		0.181	88 ± 6	24		<0.001	<0.001
BDNF	79 ± 4	22	<0.001	0.001	123 ± 5	23	<0.001	<0.001	<0.001
K252a	62 ± 3	45	0.025	0.026	84 ± 4	53	0.813	0.549	<0.001
Rapamycin	66 ± 3	37	0.004	0.001	104 ± 7	36	0.124	<0.001	<0.001
			<*0.001*				<*0.001*		
			MW-RST *ANOVA*				MW-RST *ANOVA*		

P^#^, P-value of drug treatment groups vs. untreated controls of the same hormone category; P^*^, P-value of T3^+^ vs. T3^−^ of the same drug category; P°, P-values of LD vs. HD of the same drug category. Statistical tests as in Table 1. n, number of fields.

In control cultures, GABAergic axonal length was similar in HD and LD fields in T3^−^ cultures (Figures 11A, 12B), but in T3^+^ cultures GABAergic axonal length was increased in HD fields compared with LD fields (Figures 11B, 12B, Table 6). Accordingly, the HD/LD axonal length ratio was increased in T3^+^ cultures compared with T3^−^ cultures (Figure 12B). The comparison of HD/LD ratios of control and drug-treated cultures showed that the concentration of axonal growth near cell bodies was increased in BDNF- and rapamycin-treated cultures (Figures 11C,D,G,H, 12B). In contrast, K252a-treated cultures showed a disruption of the correlation between axonal length and local cell density in T3^+^ networks (Figures 11E,F, 12B). Thus, although Rap may decrease overall axonal growth, a disruption of T3-typical effects was seen only with the blockade of trkB pathway (Figures 12A,B, Table 6).

#### GABAergic boutons

All fields considered, the number of boutons increased in T3^+^ control cultures, compared with T3^−^ control cultures (Figure 12C, Table 5). All T3^−^ drug-treated cultures showed increased counts of boutons. Of the T3^+^ drug-treated cultures, only T3^+^/K252a cultures showed increased number of boutons.

As seen above, in T3^+^ control cultures, the number of boutons was positively correlated with the number of non-GABA cell bodies present in single fields (Figure 6D), translating in a marked increase of bouton counts in T3^+^/HD fields compared with T3^+^/LD fields (Figure 6F). In T3^−^ cultures, no difference in bouton counts was detected between LD and HD fields (Figures 6D,F, 12D, Table 6). Of the drug-treated sets, both T3^+^/BDNF and T3^+^/Rap sets show a significant increase of bouton counts in the HD fields compared with LD fields (Figure 12D). In T3^+^/K252a set, no difference was present between LD and HD fields (Figure 12D).

To isolate the changes in bouton density from changes in axonal length, we calculated the number of boutons/mm axon length. An overall increase in boutons along GABAergic axons is evident after treatment with rapamycin, independent of T3 treatment (Figure 12E). Further, T3^+^ and T3^−^ control cultures showed similar HD/LD ratios, and this remained unchanged in K252a- and rapamycin-treated cultures (Figure 12F). BDNF-treated cultures, however, showed an increased HD/LD ratio in T3^+^ cultures (Figure 12F).

## Discussion

Early in cortical development, during the initial formation of synapses and appearance of early synchronized network activity, T3 promotes the GABAergic interneuron development, along with increasing synaptogenesis and network activity (Westerholz et al., 2010). These results are here extended by showing that not only T3 stimulation accelerates the formation of GABAergic boutons, but it also has profound effects on the distribution of GABAergic axons among other neurons. In the presence of T3, L-GABA axons and synapses distribute near and around groups of non-GABA, forming conspicuous nests of perisomatic boutons. These characteristic nests arise from the enhanced formation of GABAergic boutons in the vicinity of non-GABA, and apparently also from the pruning of GABAergic axons from cell-poor regions (Figure 6). Because the total axonal length in cultures lacking T3 showed no correlation with local cell density and was similar to the axonal length in T3^+^ cell-rich fields (Figures 6C,E), it is feasible that the differential axonal distribution may arise by a pruning process, rather than an axonal targeting mechanism.

The reorganization of synaptic contacts and pruning of axonal projections is an important process in the establishment and refinement of neuronal networks. Following the initial formation of synaptic connections, many developing circuits undergo a period of regression and refinement, through which some connections are eliminated while others are strengthened (Purves and Lichtman, 1980; Lohof et al., 1996; Zhang and Poo, 2001). It is typical for the initial wiring of neuronal networks that an excess of connections is formed, which are then refined by a selective, often activity-dependent pruning process (Innocenti and Price, 2005). Interestingly, thyroid hormone has been suggested to play a role in the refinement of exuberant axons (Innocenti and Price, 2005). T3 has been shown to regulate the disappearance of transient callosal projections (Lucio et al., 1997; van Welie et al., 2006). In animals made hypothyroidic during early development, callosal projections that are normally transient, could also be found in adult animals. This T3-mediated process might be activity-dependent, since the refinement of callosal projections is also regulated by neuronal activity (Wang et al., 2007a).

Early T3 action in the emerging cortical network might thus promote activity-dependent stabilization of connections between GABAergic and synchronously active projection neurons.

### T3-dependent development of GABAergic neurons and network activity: NKCC1 and KCC2 expression and the GABA switch

GABAergic signaling is not only controlled by the density and distribution of GABAergic synapses, but among other factors, it also depends on the intracellular chloride concentration in the postsynaptic cell. During the early development of the cerebral cortex, GABAergic signaling generates depolarizing potentials in the postsynaptic neurons. After the first postnatal week, the intracellular chloride concentration decreases and GABA-dependent membrane hyperpolarization appears, due to an expression change of the predominant potassium-chloride co-transporters NKCC1 and KCC2 (Lu et al., 1999; Rivera et al., 1999, 2005; Blaesse et al., 2009). The GABA shift in culture occurs between 9 and 20 DIV, and KCC2 expression increases in the same period (Figures 1, 2; see also Baltz et al. (2010)).

To examine the influence of T3 on the GABA shift, we compared in T3^+^ and T3^−^ cultures the developmental expression of NKCC1 and KCC2. Our results reveal that KCC2 expression and the GABA shift are regulated by thyroid hormone. The typical upregulation of KCC2 protein expression and the decrease of depolarizing response to muscimol are delayed in T3^−^ cultures. These results support the idea of a correlation between the upregulation of KCC2 expression and the developmental GABA shift (Rivera et al., 1999; Lee et al., 2005; Zhu et al., 2005), and concur with recent studies in the auditory brainstem and in the hippocampus (Friauf et al., 2008; Hadjab-Lallemend et al., 2010) showing that thyroid hormone signaling modulates GABAergic signaling, potentially by regulating KCC2 expression and function.

However, in 21-day-old T3^+^ and T3^−^ cultures, a significant difference in expression of KCC2 contrasts with similar response to muscimol in both culture sets (Figure 2). This might be explained by the decrease of NKCC1 expression at this time. NKCC1 promote accumulation of intracellular Cl^−^, maintaining GABA depolarizing signaling (Dzhala et al., 2005; Nakanishi et al., 2007; Blaesse et al., 2009). In line with earlier studies (Balena and Woodin, 2008; Pfeffer et al., 2009; Sipila et al., 2009), our results suggest that the decreased expression of NKCC1 suffice to prevent a depolarizing GABA response.

NKCC1 expression is activity-dependently regulated (Balena and Woodin, 2008), and is apparently regulated by T3 signaling at 14 and 21 DIV (Figure 1). T3^+^-treated cultures showed a maximum of NKCC1 expression at 14 DIV. The increase of NKCC1 might correlate with the previous emergence (at ca. 8–9 DIV) of synchronized network activity, which is partially driven by GABA depolarizing signals (Opitz et al., 2002). The decrease of NKCC1 expression between 14 and 21 DIV occurs in both T3^+^ and T3^−^ cultures, being age-dependent, probably due to increase in coherence of pre- and postsynaptic activity in the network (Balena and Woodin, 2008). Thus, our results suggest that T3 deficit disturb the coordinated KCC2 and NKCC1 expression regulation by delaying or preventing KCC2 upregulation.

Interestingly, the T3-dependent regulation of the GABA shift may affect only projection neurons. The absence of KCC2 expression upregulation in T3^−^ cultures was correlated temporally with a delayed GABAergic signaling switch only in non-GABA (Figures 1, 2). The GABA shift in GABAergic neurons might occur in a singular dynamic or not occur at all, depending on the subpopulation. Indeed, recent results also show that synapses formed by some GABAergic neurons remain depolarizing also in adulthood (Woodruff et al., 2009, 2010). Additionally, Banke and McBain (2006) showed that, in contrast to principal cells, GABAergic inputs onto a population of CA3 hippocampal interneurons did not undergo the depolarizing to hyperpolarizing shift and remained shunting throughout development.

### Temporal specificity of T3-dependent development of GABAergic neurons and cortical network activity

The most severe consequences of thyroid hormone deficits *in vivo* are restricted to the developmental period up to the end of the second postnatal week in rodents. Irreversible mental retardation and locomotor deficiencies follow cortical, hippocampal and cerebellar malformations (Oppenheimer and Schwartz, 1997; Koibuchi and Chin, 2000; Bernal et al., 2003; Bernal, 2007). Alterations in the formation of the network structure, especially those alterations concerning excitatory-inhibitory balance and the structure of the synaptic connections may explain the consequences of mature networks' malfunction. Since most T3 effects have been observed during early development, this hormone is thought to accelerate and synchronize neuronal maturation. Lack of T3 during the first 2–3 weeks of postnatal development results in a delayed upregulation of specific proteins (Royland et al., 2008; Morte et al., 2010), and a delay in network development (Venero et al., 2005; Wallis et al., 2008). If developmental processes occur out of phase, windows of opportunity may be missed and irreversible maladaptive changes may occur.

The T3 critical period (so-called because after its closure, the addition of T3 does not support recovery), coincides with the developmental period of network activity characterized by large synchronous oscillations of Ca^2+^ transients. Each neuron oscillates between periods of silence and periods of burst firing. With maturity, the duration of individual bursts decreases (Baltz et al., 2010), and the network activity pattern becomes increasingly faster and more complex, leading regionally to desynchronized patterns of activity (Allene et al., 2008; Golshani et al., 2009; Baltz et al., 2010).

In cortical networks, the excitability increased steadily with development, and the difference between bursting activity in T3^+^ and T3^−^ networks increased during the third week *in vitro*. Network-wide burst frequency and single cell frequency were decreased after any deficit of T3 during development, and the supplementation of T3 during the third week did not rescue effects of earlier T3 deprivation. Interestingly, the decreased network bursting frequency was not caused by fewer active or less participating neurons, as the attendance to single bursts was not different between T3-treated and T3-untreated networks, but by the decreased bursting activity of single neurons. Thus, T3 deprivation decreases the excitability of neurons, what may be explained by a decrease in Na^+^ currents, as showed by Dietzel and collaborators in hippocampus and cortical networks (Potthoff and Dietzel, 1997; Hoffmann and Dietzel, 2004; Niederkinkhaus et al., 2009).

The prolonged burst duration of deficient networks suggests an imbalanced AMPAR/NMDAR activity ratio. Baltz et al. (2011) showed that the duration of network bursts increases with the relative strength of NMDAR activity. In T3 deficient networks, we may have not an increased NMDAR activity, but a decreased AMPAR activity.

Morphological analyses reveal that a T3-dependent acceleration of the somatic growth of L-GABA (Figure 4E) disappeared around 21 DIV. The density of these neurons does not differ significantly between T3^+^ and T3^−^ cultures at 21 DIV, but regressive development (e.g., circuitry and synaptic trimming), which follows the period with a maximal synaptogenesis rate (Blue and Parnavelas, 1983; Ichikawa et al., 1993), may have an influence on this result. A restriction of the GABAergic neurons axonal growth may limit the soma size increase in T3^+^ cultures after 14 DIV.

During the early network developmental phase, T3^+^ networks sustain a differential distribution of GABAergic axonal endings. L-GABA grow synaptic boutons preferentially in regions rich in cell bodies of projection neurons, sparing cell body-free areas. This regionally differentiated innervation is significantly reduced in T3^−^ cultures (Figure 6). The deficit in bouton innervation is maintained during the third week *in vitro* despite the presence of T3 (Figure 7).

T3 promotes the expression of KCC2 and the development of the GABAergic synaptic network during the early period characterized by the presence of pronounced synchronous network activity. The characteristic T3-dependent GABAergic innervation of non-GABAergic cell bodies was reduced in cultures lacking T3 during the first 2 weeks *in vitro*, suggesting that an early deficit causes permanent damage in the network architecture. Our results corroborate the idea that T3 accelerates neuronal maturation during the early network development, and by doing so, contribute to the timely synchronization of critical aspects of network activity and GABAergic system development.

### T3-dependent development of GABAergic neurons and network activity: the role of trKB and mTOR signaling pathways

Early T3 actions contributing to the maturation of the excitatory-inhibitory balance might be regulated by activity-dependent expression of neurotrophic factors. The neurotrophin BDNF is well-known as a mediator of activity-dependent effects: Its secretion by projection neurons in the cerebral cortex is regulated by neuronal activity (Zafra et al., 1990; Lessmann et al., 2003). Additionally, BDNF strongly promotes interneuron development (de Lima et al., 2004; Palizvan et al., 2004; Patz et al., 2004; Woo and Lu, 2006), regulating GABAergic signaling at different levels (Aguado et al., 2003; Wardle and Poo, 2003; Jovanovic et al., 2004). Moreover, BDNF transcription is regulated by thyroid hormone (Neveu and Arenas, 1996; Luesse et al., 1998; Koibuchi et al., 1999, 2001; Camboni et al., 2003; Royland et al., 2008; Sui et al., 2010; Chakraborty et al., 2012; Gilbert and Lasley, 2013).

Although the T3-mediated increase of L-GABA soma size at 14 DIV seems independent of BDNF signaling, other aspects of T3-mediated changes in GABAergic neurons development are clearly regulated by BDNF. The addition of trkB inhibitor K252a to T3^+^ cultures decreased or abolished the positive correlation between axonal parameters and local circuitry (Figures 11, 12). Both the reduction of GABAergic axonal growth in LD fields and the characteristic increase of boutons in high density fields were drastically reduced by the trkB receptor inhibitor K252a (Figure 12). Bouton density, however, is not, as expected, decreased in high density fields, but instead it is higher in low density fields. This result suggests that BDNF signaling not only promote the increase of boutons in high density fields, but also decreases bouton formation in low density fields.

After application of exogenous BDNF to T3-deficient networks, axonal parameters showed an increased correlation with circuitry (Table 6). Notably, BDNF increased GABAergic bouton formation in T3^−^ cultures (Figure 12C). In other cases BDNF action was not sufficient to mimic control conditions in the presence of T3. The interpretation of tests in the absence of T3 should take in account that unoccupied T3 receptors do show aberrant activity and interfere in complex ways with the developmental program (Morte et al., 2002; Bernal, 2007). Conversely, in the presence of T3, exogenous BDNF increased the total length of GABAergic axons and the density of boutons in high density fields, thus increasing considerably the difference of these variables in high and low density fields (Figures 12B,D,F, Table 6).

Because expression of BDNF is also modulated by neuronal activity, BDNF signaling may differentially increase T3 actions in active local networks and contribute to the regional and temporal heterogeneity of T3 function (Gereben et al., 2008; Williams and Bassett, 2011). Beyond the intrinsic developmental program, both GABAergic interneurons and BDNF are implicated in the developmental plasticity of sensory-related connections. It is then feasible to hypothesize that related actions of T3 in the sensorial pre-critical period might have profound consequences for the appropriate development of sensory-motor system.

The serine/threonine protein kinase mTOR regulates survival, differentiation and development of neurons (for review see Swiech et al., 2008). Inhibition of mTOR by rapamycin decreases neurite outgrowth, cell size and neuronal differentiation markers (e.g., Zeng and Zhou, 2008). Rapamycin also suppresses growth cone formation (Verma et al., 2005), axonal sprouting (Buckmaster et al., 2009; Buckmaster and Wen, 2011), and outgrowth (Okada et al., 2011) in a variety of models (Crino, 2011). Interestingly, also synaptogenesis and synaptic plasticity are thought to be regulated by mTOR (Bourgeron, 2009; Hoeffer and Klann, 2010; Li et al., 2010).

Our results show that rapamycin decreases excitability and synchronized calcium activity in cortical networks (Figure 8). This concurs with previous results (Daoud et al., 2007; Wang et al., 2007b; Li et al., 2008; Zeng et al., 2009; Huang et al., 2010; Moavero et al., 2010; Buckmaster and Lew, 2011; Buckmaster and Wen, 2011; Crino, 2011; Feliciano et al., 2011) showing increased excitatory synaptic current durations and epileptiform patterns with increased mTOR signaling (Wang et al., 2007b; Zeng et al., 2009). Li et al. (2008) specifically showed that rapamycin reduces synchronized calcium spikes in cultured hippocampal networks. The rapamycin-dependent decrease of excitability in young networks corroborates the idea that mTOR mediates T3 effects on the network activity.

Here we also show that rapamycin decreases GABAergic axonal length, suggesting that mTOR signaling promotes GABAergic axonal growth (Figure 12A), and that T3 effects on axonal growth depend on the mTOR signaling level. As rapamycin treatment decreases axonal growth and increases GABAergic presynaptic bouton density, it is possible that mTOR signaling favors GABAergic neuritic extension rather than synaptogenesis. Other recent studies showed specific mTOR modulation on GABAergic neurons (Fu et al., 2012). TSC1 knockout or knockdown mutants, with the typical mTOR signaling hyperactivity, show enlarged GABAergic neurons in the cortex and hippocampus. Additionally, numbers of specific GABAergic neuronal types are reduced, probably due to impaired interneuronal migration.

mTOR not only mediates T3 actions (Moeller et al., 2006; Sui et al., 2008), but apparently also mediates BDNF effects on neuronal plasticity (Schratt et al., 2004; Inamura et al., 2005; Liao et al., 2007; Zhou et al., 2010). Thus, it is possible that these pathways interact, when T3-dependent changes of axonal growth are modified by both trkB and mTOR signaling. Alternatively, the overlap of action of mTOR signaling and BDNF signaling on the regulation of axonal length and bouton density might relate to local network activity changes. In this view, primary T3-mediated changes on network activity by, for example, modulating ion channels or transporters expression (Hoffmann and Dietzel, 2004; Friauf et al., 2008; Niederkinkhaus et al., 2009), may secondarily regulate the GABAergic development.

Some changes triggered by blockade of mTOR function are not similar to the effects of BDNF blockade. Some T3-dependent GABAergic development aspects are regulated either by mTOR- or by trkB signaling (cell size or population changes vs. bouton density), suggesting the existence of parallel regulatory pathways for T3-dependent changes. This agrees with the idea of multigenic mechanisms determining T3 actions (Nunez et al., 2008).

## Authors contributions

Conceived and designed the experiments: Sören Westerholz, Ana D. de Lima, and Thomas Voigt. Performed the experiments: Sören Westerholz, Ana D. de Lima, and Thomas Voigt. Analyzed the data: Sören Westerholz and Ana D. de Lima. Wrote the paper: Sören Westerholz, Ana D. de Lima, and Thomas Voigt.

### Conflict of interest statement

The authors declare that the research was conducted in the absence of any commercial or financial relationships that could be construed as a potential conflict of interest.

## Abbreviations

BDNF: brain derived neurotrophic factor
DIV: Days *in vitro*
HD: high cell density (fields)
KCC2: K^+^-Cl^−^ cotransporter 2
LD: low cell density (fields)
L-GABA: large GABAergic neuron
mTOR: mammalian target of rapamycin
MW-RST: Mann-Whitney Rank Sum Test
NKCC1: Na^+^-K^+^-Cl^−^ cotransporter
non-GABA: non-GABAergic neuron
Rap: rapamycin
S-GABA: small GABAergic neuron
T3: triiodothyronine, trkB, tropomyosin-receptor-kinase B.
